# Society for Immunotherapy of Cancer consensus statement on immunotherapy for the treatment of bladder carcinoma

**DOI:** 10.1186/s40425-017-0271-0

**Published:** 2017-08-15

**Authors:** Ashish M. Kamat, Joaquim Bellmunt, Matthew D. Galsky, Badrinath R. Konety, Donald L. Lamm, David Langham, Cheryl T. Lee, Matthew I. Milowsky, Michael A. O’Donnell, Peter H. O’Donnell, Daniel P. Petrylak, Padmanee Sharma, Eila C. Skinner, Guru Sonpavde, John A. Taylor, Prasanth Abraham, Jonathan E. Rosenberg

**Affiliations:** 1 University of Texas MD Anderson Cancer Center, 1515 Pressler Unit 1373, Houston, TX 77030 USA; 2 Dana-Farber Cancer Institute, Brookline, MA 02446 USA; 3 Tisch Cancer Institute at Mount Sinai Medical Center, New York, NY 10029 USA; 4 University of Minnesota, Minneapolis, MN 55455 USA; 5 BCG Oncology, Phoenix, AZ 85032 USA; 6 Bladder Cancer Advocacy Network, North Carolina Triangle Chapter, Chapel Hill, NC 27517 USA; 7 The Ohio State University Wexner Medical Center, Columbus, OH 43210 USA; 8 University of North Carolina, Chapel Hill, NC 27599 USA; 9 University of Iowa, Iowa City, IA 52242 USA; 10 University of Chicago, Chicago, IL 60637 USA; 11 Yale Cancer Center, New Haven, CT 06520 USA; 12 University of Texas MD Anderson Cancer Center, Houston, TX 77030 USA; 13 Stanford University, Stanford, CA 94305 USA; 14 University of Alabama, Birmingham, AL 35294 USA; 15 University of Kansas Cancer Center, Kansas City, KS 66160 USA; 16 University of Texas MD Anderson Cancer Center, Houston, TX 77030 USA; 17 Memorial Sloan Kettering Cancer Center, New York, NY 10065 USA

**Keywords:** Bladder cancer, Immunotherapy, Guidelines, Consensus statement

## Abstract

The standard of care for most patients with non-muscle-invasive bladder cancer (NMIBC) is immunotherapy with intravesical Bacillus Calmette-Guérin (BCG), which activates the immune system to recognize and destroy malignant cells and has demonstrated durable clinical benefit. Urologic best-practice guidelines and consensus reports have been developed and strengthened based on data on the timing, dose, and duration of therapy from randomized clinical trials, as well as by critical evaluation of criteria for progression. However, these reports have not penetrated the community, and many patients do not receive appropriate therapy. Additionally, several immune checkpoint inhibitors have recently been approved for treatment of metastatic disease. The approval of immune checkpoint blockade for patients with platinum-resistant or -ineligible metastatic bladder cancer has led to considerations of expanded use for both advanced and, potentially, localized disease. To address these issues and others surrounding the appropriate use of immunotherapy for the treatment of bladder cancer, the Society for Immunotherapy of Cancer (SITC) convened a Task Force of experts, including physicians, patient advocates, and nurses, to address issues related to patient selection, toxicity management, clinical endpoints, as well as the combination and sequencing of therapies. Following the standard approach established by the Society for other cancers, a systematic literature review and analysis of data, combined with consensus voting was used to generate guidelines. Here, we provide a consensus statement for the use of immunotherapy in patients with bladder cancer, with plans to update these recommendations as the field progresses.

**Electronic supplementary material:**

The online version of this article (doi:10.1186/s40425-017-0271-0) contains supplementary material, which is available to authorized users.

## Background

Despite a slight but steady decrease in incidence and deaths from bladder cancer over the past few decades in the U.S., an estimated 79,000 people will be newly diagnosed in 2017, and nearly 17,000 will die from the disease [1]. These data underscore the need for novel treatment strategies to improve patient outcomes. As understanding of the role of the immune system in the pathogenesis of cancer has advanced, there has been increasing interest in treatments that rely on immunomodulatory mechanisms to target and destroy cancer cells. Such agents, which include cytokines, monoclonocal antibodies, immune checkpoint inhibitors, T cell therapies, oncolytic viruses and vaccines, have allowed a subset of patients to benefit from durable response rates, often with a more tolerable adverse event profile than traditional therapies [2]. The need to identify why certain patients respond to a given therapy when others fail to achieve measurable clinical benefit has led to energetic efforts to identify and validate predictive biomarkers that can guide patient selection, and prognostic biomarkers to help evaluate likely disease outcomes. Other key areas of interest include rational combination therapies and drug sequencing, and the potential role of systemic immunotherapy to treat organ-confined and early disease.

**Table 1:** Ongoing Selected Immunotherapy Trials in Bladder Cancer

*Completed*		
Drug/Agent	Study	Stage of Disease
Atezolizumab (MPDL3280A) Anti-PD-L1 Cohort 1 NCT02951767, Cohort 2: NCT02108652	Phase II	Locally advanced or metastatic – progressed after platinum-based treatment (Rosenberg et al. *Lancet* 2016; Balar et al. *Lancet* 2016 [63, 74])
Pembrolizumab NCT02256436	Phase III vs. standard of care chemotherapy	Locally advanced or metastatic – progressed after platinum-based treatment
*Ongoing*		
Drug/Agent	Study	Stage of Disease
Durvalumab with or without tremelimumab NCT02516241	Phase II vs. standard of care chemotherapy	Stage IV transitional cell carcinoma of the urothelium
Atezolizumab NCT02662309	Phase II preoperative MPDL3280A	Transitional cell carcinoma of the bladder
Atezolizumab combination with cisplatin and gemcitabine NCT02989584	Pilot safety, single-arm phase II study	Metastatic bladder cancer
Atezolizumab NCT02450331	Randomized phase III atezolizumab as adjuvant therapy vs. observation	PD-L1 positive, high risk muscle invasive bladder cancer
Nivolumab NCT02632409	Randomized phase III nivolumab as adjuvant therapy vs. placebo	High risk muscle-invasive bladder cancer
Maintenance avelumab NCT02603432	Phase III vs. best supportive care alone	Locally advanced or metastatic bladder cancer that did not progress after completion of first-line platinum containing chemotherapy
Pembrolizumab NCT02335424	Phase II	Non-cisplatin eligible patients
MEDI-4736 (anti-PD-L1) +/− tremelimumab (anti-CTLA-4) NCT02516241	Phase III, three arms: MEDI-4736 +/− tremelimumab vs. standard of care chemotherapy	Unresectable stage IV bladder cancer

Given the immunological nature of the standard treatment approach to bladder cancer, which relies on intravesical instillation of Bacillus Calmette-Guérin (BCG), a live, attenuated strain of *Mycobacterium bovis*, there is a clear rationale for expanded use of immune-based treatments for bladder cancer. In order to enable clinicians to understand and use the increasing number of emerging immunotherapies effectively and safely, the Society for Immunotherapy of Cancer (SITC) convened a Task Force of experts on bladder cancer, including physicians, patient advocates, and nurses, to address issues related to patient selection, toxicity management, clinical endpoints, and sequencing and combination of therapies. This panel met initially in December 2014 with the goal of generating consensus recommendations for the clinical use of immunotherapy for bladder cancer. Discussion at this meeting centered on the various consensus papers recently published, and how these might be refined, or variations better explained [3].

The following objectives were deemed priorities for Task Force discussion: 1) To determine consensus on a) the definition of risk categories and b) identification of patients for whom intravesical immunotherapy is appropriate, 2) to determine consensus around duration of intravesical therapy and maintenance vs. non-maintenance regimens, 3) to determine which patients this approach is not appropriate for (i.e., patients with very low risk of progression or recurrence), 4) to define the timing of additional trans-urethral resection in conjunction with BCG therapy and procedures to reduce toxicity without impacting efficacy, and 5) to define the role of immune checkpoint blockade for metastatic disease. Following the in-person meeting, the Task Force continued to address recent advances in the field through telephone and email communications. A commentary section is provided that addresses some of these issues.

### Non-muscle invasive bladder cancer (NMIBC)

NMIBC (previously commonly referred to as “superficial” bladder cancer) is the most common presentation of urothelial cancer [4]. The treatment of NMIBC, which depends on risk stratification based on clinical and pathologic criteria, largely relies on transurethral resection followed by intravesical instillation of therapy, primarily with BCG immunotherapy [5, 6] or chemotherapy. BCG is currently the treatment of choice for urothelial carcinoma in situ (CIS), since it has been shown to reduce risk of recurrence, and of progression of NMIBC after transurethral resection [7]. Although the mechanisms that underpin the efficacy of intravesical BCG are incompletely understood, it is widely believed that immune infiltration is essential to an effective response [8] and that both urothelial cells and bladder cancer cells contribute to the overall antitumor effect [9]. Several consensus panels and guidelines have been developed in the past few years to identify and categorize the appropriate patients to undergo intravesical therapy [10–16]. Characteristics used for prognostication and to guide treatment include histologic grade, number of tumors, prior recurrence patterns, extent of disease, and, if present, carcinoma in situ. Guidelines to date, while similar, have areas of controversy, which imply a need to further define prognostic criteria and optimal management, particularly with respect to intermediate risk patients [17, 18].

### Muscle invasive and advanced bladder cancer

Two randomized clinical trials as well as meta-analyses demonstrate a survival benefit with the integration of neoadjuvant cisplatin-based chemotherapy prior to cystectomy for patients with muscle-invasive disease. Adjuvant cisplatin-based chemotherapy has never been definitively proven to improve overall survival, but it is frequently used. Systemic therapy for metastatic disease has historically consisted of platinum-based chemotherapy, and this is discussed in the consensus report [19, 20]. The following objectives were deemed as priorities for the discussion of the panel meeting for muscle invasive and advanced disease: 1) to define the role of immune checkpoint inhibitors in the treatment of metastatic urothelial cancer, 2) to comment on the clinical utility of biomarkers predictive of benefit to treatment with these therapies, and 3) to discuss potential utility of immune-based therapy in the muscle invasive, non-metastatic setting.

## Methods

### Consensus statement policy

This consensus statement utilized the National Academy of Medicine’s (NAM, formerly the Institute of Medicine) Standards for Developing Trustworthy Clinical Practice Guidelines reported in March 2011 [21]. In addition, the previously released SITC consensus guidelines were used as a model to develop and organize this manuscript as previously described [22, 23]. As outlined by the NAM, the development of clinical practice guidelines should include a transparent process. This includes information regarding the development of guidelines, funding sources, and the reporting and management of conflicts of interest. Moreover, the Task Force nominated to develop guidelines should be a multi-disciplinary group and base their recommendations on evidence in the literature with a rating system to evaluate the strength of supporting peer-reviewed literature and results from clinical trials reported.

To develop these guidelines, SITC sponsored a panel led by a steering committee of bladder cancer experts who met in December 2014 in person. To discuss updates to the field, the panel subsequently communicated via email. The meeting and follow-up discussions were guided with the goal of developing clinical treatment guidelines specifically for immunotherapy in bladder cancer patients. This consensus statement is only intended to provide guidance; it is not to be used as a substitute for the individual professional judgment of the treating physician. The full version of this consensus report and others can be found on the SITC website [23]. Because of differences in drug approval, availability, and regulations in other countries, the panel focused on drugs currently approved by the U.S. Food and Drug Administration (FDA) for the treatment of patients in the U.S.

### Consensus panel and conflicts of interest

In accordance with the practices utilized in previous SITC consensus guidelines, panel members were both SITC members or nonmembers but represented multiple disciplines, including patient representatives, nurses, and others expected to be affected by the development of clinical practice guidelines. All Task Force members were required to disclose any conflicts of interest using the SITC disclosure form, which requires full financial and other disclosures concerning relationship with commercial entities that could be expected to have direct regulatory or commercial impact resulting from the publication of this statement. An advanced copy of this manuscript was available for comment by SITC membership prior to publication (Additional file 1). No commercial funding was provided to support the consensus panel, literature review, or the preparation of this manuscript.

### Bladder cancer consensus task force

The Task Force consisted of 17 participants, including 8 medical oncologists, 7 urologists, 1 nurse, and 1 patient representative (Additional file 2). The urologists were chosen for their experience in the development and evaluation of best practice guidelines for the use and optimization of BCG therapy, and all members were experts in the management of the spectrum of urothelial cancer. The medical oncologists were experienced in the management of advanced bladder cancer with both chemotherapy and immunological therapy, including participation in clinical trials of immune checkpoint inhibitors. Additional participants were experts in addressing issues of barriers in access to appropriate use of immunotherapy. A list of the Task Force pre-meeting survey questions and responses is available in Additional file 3.

### Literature review

The MEDLINE database was used to perform the literature search by combining the terms “transitional cell carcinoma OR bladder cancer OR urothelial cancer” AND “BCG,” “interferon” “ipilimumab.” The search was limited to clinical trials, meta-analyses, practice guidelines, and research in humans. The original search leading up to the meeting encompassed articles published 2006–2014 (conducted on November 21, 2014). The literature search was updated on June 12th, 2017 to include more recent publications from the original search and to add terms reflecting recent advances in the field. The updated bibliography was generated by re-running the original search for years 2015–2017, and supplementing this with search results for “transitional cell carcinoma OR bladder cancer OR urothelial cancer” in combination with “nivolumab”, “pembrolizumab”, “atezolizumab”, “durvalumab”, “avelumab”, “PD-1 or PD-L1”, “combination therapy” and “peptide-derived vaccine”. Date limits for this aspect of the search were 2010–2017. After removing duplicates, reviewing the references for accuracy, and supplementing with additional references as identified by the consensus panel, the updated bibliography resulted in a 213-item list (Additional file 4). Using the previously established grading system, the supporting literature was graded into three levels [22]. To summarize, Level A was defined as strong, evidence-based data derived from prospective, randomized clinical trials and meta-analyses. Level B literature consisted of moderately supported data from uncontrolled, prospective clinical trials. Level C represented weak supporting data derived from reviews and case reports.

## Consensus recomendations

### What is the role of BCG therapy in non-muscle invasive bladder cancer (NMIBC)? Specifically, when should intravesical immunotherapy be used among the various risk categories of NMIBC?

The literature and multiple consensus statements report slightly different recommendations for management of different risk categories of bladder cancer. However, all agree that risk-stratification is key to treatment recommendations. Additionally, the consensus reports all describe significant benefit that can be experienced by reduction in recurrence and progression. This is also stated in guidelines by the American Urologic Association (AUA), the European Association of Urology (EAU), and the International Bladder Cancer Group (IBCG) [10–16].

### Is there a role for BCG in high risk (high grade) bladder cancer?

#### Literature review and analysis

High risk NMIBC is defined in most consensus reports as histologically confirmed high grade tumor (including Ta and T1 tumors) as well as carcinoma in situ [24]. High risk could also include certain large volume low grade tumors, although most experts now would consider these as intermediate risk tumors. Consensus statements from several urologic and bladder cancer groups (AUA, EUA, IBCG, NCCN, ICUD) recommend induction BCG for all high risk tumors, with differing recommendations for maintenance BCG [10–16].

BCG induction (6 weeks treatment) followed by 3 week maintenance BCG has significant beneficial impact on disease recurrence, progression, and outcomes, with superior results relative to chemotherapy [25]. Several randomized trials have demonstrated this and are summarized in Kamat et al. [14]. The final report from the study EORTC 98013, in which the dose (administered at three weekly instillations at months 3, 6, 12, 18, 24, 30, and 36 according to the SWOG schedule) and duration (1 year versus 3 years) of BCG maintenance were tested, demonstrated that full-dose BCG maintenance is more effective without added toxicities than the one-third dose at the same schedule. In addition, patients with high risk disease benefited from 3 years of maintenance [26]. A recently completed Spanish Oncology Group (CUETO) study, in which the BCG maintenance therapy was modified to one instillation every 3 months, did not show a benefit of maintenance [27], and this has also been seen in a number of reports utilizing modified approaches to maintenance BCG [14]. Additionally, EORTC conducted a trial comparing BCG maintenance (SWOG schedule) to epirubicin maintenance and demonstrated significant superiority of BCG compared with epirubicin for all clinical parameters (time to first recurrence, time to distant metastases, and disease-specific as well as overall survival) in patients with both high and intermediate risk disease [28].

A recently published European phase III trial compared chemohyperthermia using mitomycin C (MMC) versus BCG as adjuvant therapy for intermediate and high risk patients [29]. Patients were accrued over 10 years, but there were still small patient numbers (*n* = 190). Thus, the study was closed early and was underpowered. However, the results have piqued interest, in that those who were treated per protocol had a significantly improved 24 month recurrence-free survival following chemohyperthermia compared with BCG alone (*p* = 0.02). However, there was no significant difference if analyzed by intent to treat (*p* = 0.08), and 3 week maintenance BCG was given for only one year rather than the recommended 3 years [29].

#### Consensus recommendations

**Fig. 1:**
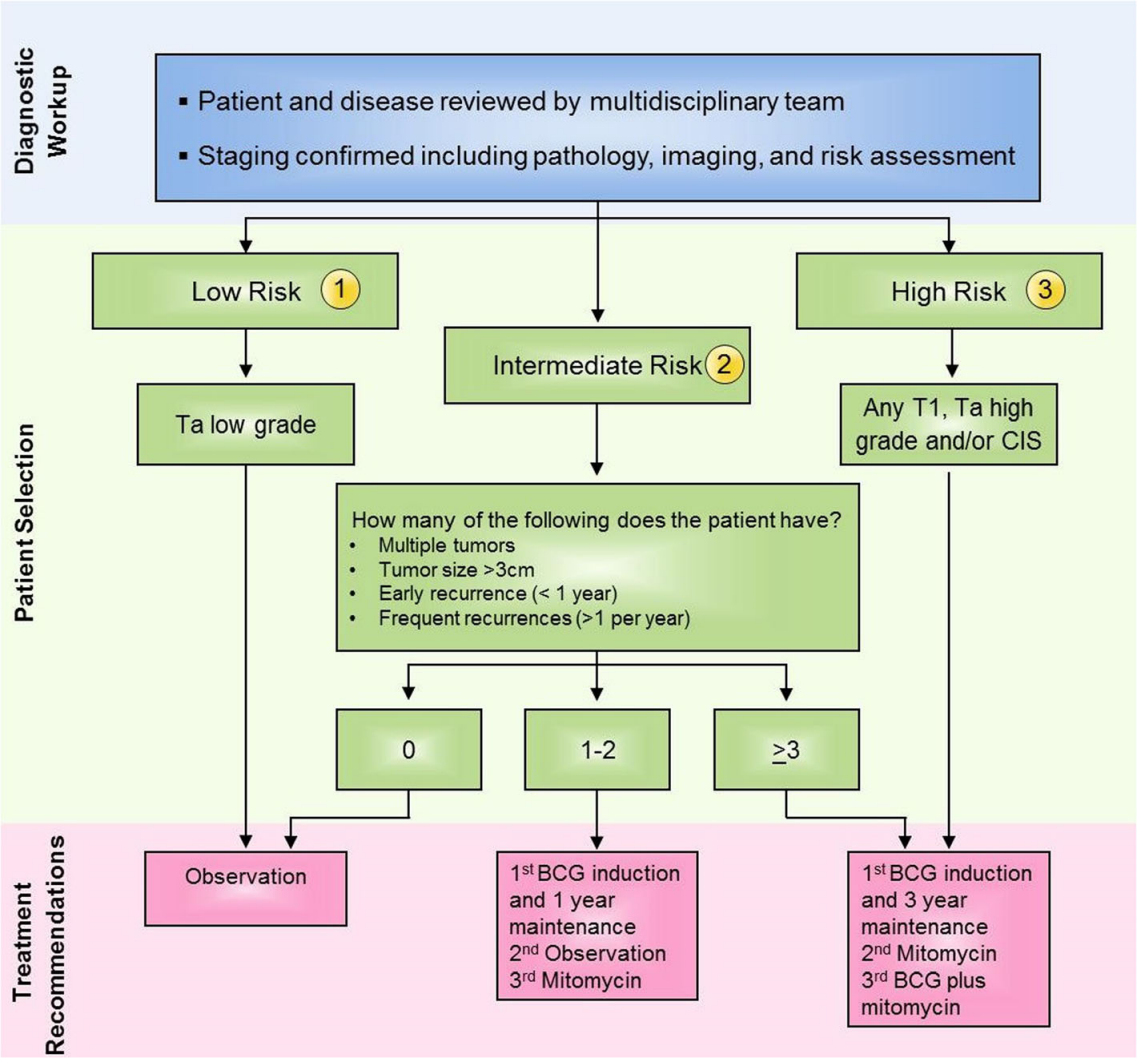
Treatment algorithm for non-muscle invasive bladder cancer. All of the treatment options shown may be appropriate. The selection of therapy should be individualized based on patient eligibility and the availability of the therapy at the discretion of the treating physician. These algorithms are meant to provide advice as the consensus recommendations of the Task Force. (1) The Task Force defines Low Risk as solitary, primary low-grade Ta tumor. (2) Intermediate Risk is defined as histologically-confirmed multiple and/or recurrent low-grade Ta tumors. (3) High risk is defined as any T1, high-grade and/or carcinoma in situ

Based on guidelines reflecting results of randomized clinical trials, the Task Force unanimously recommended that BCG therapy for high risk patients should be considered standard of care for this patient category (Fig. 1). However, the definition of high risk patient subgroups continues to be refined. Although maintenance BCG will be discussed below, it appears to be critical for successful management of high risk patients. In addition, the SWOG schedule is reproducibly providing the best efficacy. This recommendation is based on Level A evidence from randomized studies over several years [10–16, 25, 26].

### What is the role of BCG in carcinoma in situ of the bladder?

#### Literature review and analysis

Carcinoma in situ is considered high risk disease and, in most guidelines, the recommendation is for treatment with BCG, including both induction and maintenance based on randomized trials [10–15, 17, 18]. However, some would recommend the use of intravesical chemotherapy first and BCG at recurrence. One study that forms the basis of this approach reported long-term results of a randomized trial with a subset of 68 patients with carcinoma in situ [30]. The treatment consisted of mitomycin induction followed by maintenance of monthly alternating instillations of mitomycin and BCG versus mitomycin alone for up to 2 years [30]. No significant difference was found between the two groups, but the non-stratified risk of dying from bladder cancer was low overall at 28% at 15 years, with a follow-up of living patients of 17 years [30]. The EORTC study 30,993 was a randomized phase II trial of 96 patients with carcinoma in situ, comparing sequential mitomycin and BCG with BCG alone [31]. The endpoints included complete response at first cystoscopy 16–18 weeks after start of treatment, as well as disease-free and overall survival. Complete responders received maintenance on their treatment arm every 3 months for up to 3 years. The complete response and disease-free rates were similar in both groups [31]. Another approach that has been reported, but not widely adopted, is sequential BCG followed by electromotive mitomycin, particularly for high risk patients and carcinoma in situ [32]. Although sequential treatment of BCG and electromotive mitomycin C did show efficacy, challenges with its tolerability were reported [32].

#### Consensus recommendations

BCG immunotherapy is recommended in most guidelines for carcinoma in situ of the bladder, as it is a high risk category, and this was also the recommendation by the Task Force. Induction therapy with mitomycin was also discussed as an alternative to BCG. The Task Force recommendations in order of preference were as follows: BCG induction and maintenance for 3 years as per SWOG schedule, combination of BCG and mitomycin, and trial of mitomycin first with BCG reserved for those who fail chemotherapy. The Task Force also recognizes that a large majority of patients with carcinoma in situ present with papillary tumors as well, and therefore, recognizes that the majority of patients will end up being treated with induction and maintenance BCG primarily, rather than primary chemotherapy.

### What is the role of BCG in intermediate risk bladder cancer?

#### Literature review and analysis

The IBCG has recently defined intermediate-risk disease as multiple or recurrent low-grade Ta tumors and provided guidance on further stratifying these patients into categories of lower versus higher risk of recurrence or progression based on key factors, including histologic grade, centricity of tumors, size of tumors, and rate of recurrence following resection [17]. The IBCG proposes that the following factors be considered to aid in clinical decisions in intermediate risk disease: number (>1) and size of tumors (> 3 cm), timing (recurrence within 1 year), frequency of recurrences (> 1 per year), and previous treatment. In patients without these risk factors, a single, immediate instillation of chemotherapy is advised. In those with 1–2 risk factors, induction BCG with maintenance or additional intravesical chemotherapy are recommended, and previous intravesical therapy should be considered when choosing between these therapies. For those with 3–4 risk factors, induction plus maintenance BCG is recommended [17]. Treatment recommendations reflect the spectrum of the disease and do vary among groups [10–18]. Based on early results of EORTC 30911 in 500 intermediate risk patients, BCG induction with 3-week maintenance utilizing the SWOG schedule, had significant beneficial impact on disease recurrence, progression, and outcomes [14, 28]. Similarly, intermediate risk patients were included in EORTC 98013 and demonstrated benefit similar to the high risk patients. However, it was recommend to treat these patients at full dose for 1 year rather than for 3 years [27]. Again, the recent publication reporting chemohyperthermia should also be taken into consideration [29].

#### Consensus recommendations

The Task Force discussed risk stratification at length and agreed that there were varying definitions of intermediate risk. However, most felt that that most of these patients (other than those with none of the aforementioned risk factors) would benefit from BCG based on Level A evidence from randomized clinical trials. The Task Force unanimously advocated for risk stratification as a basis for deciding therapy and recommend that the risk category for the tumors be carefully assessed and the transition from low risk to intermediate risk be carefully defined. The EORTC 30911 study comparing 3-week maintenance BCG and epirubicin chemotherapy found that intermediate risk patients had even a greater reduction in metastasis and mortality than high risk patients [28]. Thus, the Task Force recommends that BCG induction and at least 1 year of maintenance therapy be used for patients with intermediate risk tumors.

### Is there a role of BCG in low risk bladder cancer?

#### Literature review and analysis

Low grade NMIBC that occur for the first time are considered low risk NMIBC. Although patients with low risk NMIBC have been shown in randomized studies to benefit from BCG in terms of risk reduction [16], its use must be weighed against the potential for side effects. In general, consensus guidelines do not recommend the use of BCG for these low risk tumors (i.e., small, solitary, superficial low-grade tumors [Ta]) [4–10]. The EAU and the AUA suggest consideration of the use of BCG when low grade tumors are large, multifocal, and/or recurrent (i.e., when these tumors fall into the intermediate risk category [Fig. 1]) [10–16]. It has been noted that BCG can be less effective in low grade tumors, presumably because they are less antigenic [33].

#### Consensus recommendations

The Task Force unanimously recommended that low risk patients (solitary, first time with low grade tumors) should not receive BCG (Fig. 1).

### What is the role of maintenance BCG?

#### Literature review and analysis

All guidelines recommend induction and maintenance BCG of 1–3 years for high risk patients with a risk reduction in terms of recurrence [10–18]. However, the ICUD guidelines only include maintenance BCG for carcinoma in situ, not for Ta high grade tumors [14]. This differs from the recommendations of AUA, EUA, and IBCG. As reviewed in previous sections, BCG induction and maintenance has been shown to be beneficial in patients with high risk and intermediate risk groups utilizing the SWOG schedule [14, 24–28]. Modifications in terms of reduction in dose or in the number of doses per session of maintenance have not been shown as beneficial [14, 27, 28]. Again, an improved definition of the patient subgroups who would benefit continues to be a topic of active clinical research. The report of EORTC 98013 suggests that 1 year of maintenance utilizing the SWOG schedule is sufficient for intermediate risk patients [27]. However, recurrence directly correlated with the duration of maintenance, with 3-year maintenance resulting in fewer recurrences in each dose group.

#### Consensus recommendations

The members of the Task Force had different opinions on this issue. However, it was agreed upon that all high risk (high grade) patients should receive maintenance therapy for 3 years, while intermediate risk patients should receive maintenance therapy for at least 1 year based on Level A evidence.

### What is the optimal timing and schedule of post-resection immunotherapy for NMIBC?

#### Literature review and analysis

Most guidelines recommend intravesical immunotherapy be initiated after an interval of at least two weeks following transurethral resection or biopsy of the bladder to avoid systemic absorption [10–16], unless repeat resection is to be performed (at 4–6 weeks as recommended for all high grade T1 patients and selected high grade Ta patients [per EAU guidelines]). Unlike chemotherapy, BCG should never be administered within 24 h of bladder tumor resection and can in fact be dangerous. Non-randomized studies show no advantage of early administration. There are no randomized data suggesting an optimal time to first dose (2 to 4 weeks). Additionally, patients who tolerate 6 weeks of BCG induction and are at high risk for tumor recurrence and progression should be treated with maintenance BCG using the SWOG schedule: 3 weekly instillations at 3, 6, 12, 18, 24, 30, and 36 months [10–16, 25].

#### Consensus recommendations

The Task Force agreed with the recommendation to wait at least 2 weeks before instillation of BCG after resection of tumor(s) based on Level A evidence. In addition, the Task Force agrees with the 6 + 3 schedule (also known as the ‘Lamm’ or ‘SWOG’ schedule) of maintenance BCG administration based on Level A Evidence.

### What is the recommended initial and maintenance dose of BCG?

#### Literature review and analysis

Based on clinical trials and clinical experience, the initial course should be 1 vial of BCG (TICE® is 50 mg; Theracys® is 81 mg) usually containing approximately 5 × 10^8^ or more CFU (the amount present in vials approved for intravesical instillation) weekly for 6 weeks [25]. This is accepted by the AUA, EUA, and the IBCG [10–16].

Increasing side effects may be reduced by serial reductions in BCG dose; most recommended dose reductions are at one-third, one-tenth, one-thirtieth, and one-one hundredth [26]. Randomized clinical trials have reported conflicting results regarding the efficacy and improved safety of dose reduction. The highly cited randomized trial by Oddens et al. showed efficacy in the following order: full dose for 3 years, one-third dose for 3 years, full dose for one year, and lastly one-third dose for one year [26].

#### Consensus recommendations

The Task Force recommended full doses for induction and the dose reduction during maintenance if necessary based on side effects, which is based on Level A evidence. The Task Force did concede that during times of BCG shortage, as has happened in recent times, it is acceptable to start induction with one-third dose if this allows a vial of BCG to be split among 3 patients to allow more patients to receive BCG than if this were not done.

### What are contraindications to the administration of BCG?

#### Literature review and analysis

Instillation of BCG in the presence of gross hematuria can result in systemic absorption and toxicity from BCG. Thus, BCG should not be instilled in the presence of gross hematuria or active urinary infection. Treatment of ongoing urinary tract infections prior to BCG instillation may reduce toxicity. In a study in which patients with high-risk NMIBC received induction intravesical BCG, 61/243 had significant bacteriuria in voided urine prior to starting therapy. In this study, asymptomatic bacteriuria did not appear to increase side effects or risk of BCG toxicity and had no impact on recurrence rates in infected patients [34]. Although BCG has been cited as contraindicated for immunologically compromised patients with bladder cancer, a retrospective study reported on 45 immunosuppressed high risk NMIBC patients treated with intravesical BCG. Of these patients, 12 had functioning organ transplants, 23 were undergoing chemotherapy for unrelated cancers, and 10 were taking steroids for autoimmune or related diseases. Although this study was conducted in a small patient population, these results suggest that BCG can be safely administered to select patients who are immunosuppressed. However, efficacy may be limited, as individuals receiving immunosuppression following organ transplantation were less likely to respond [35].

#### Consensus recommendations

Although BCG should not be administered in the presence of active infection or gross hematuria, the Task Force agreed that asymptomatic bacteriuria did not appear to increase toxicities or risk thereof based on Level B evidence. In addition, BCG appears to be safe and effective in select patients who are immunosuppressed based on small cohort studies (Level C evidence).

### What is the value of using oral quinolones following BCG administration?

#### Literature review and analysis

Administration of oral quinolones can reduce toxicity from BCG therapy and should be routinely considered in all patients undergoing intravesical BCG therapy. These data are based on two randomized clinical trials, illustrating that instillation can result in up to 20% reduction in side effects from BCG therapy [36, 37]. It is important to note that quinolones should not be administered prior to or within 6 h of BCG instillation, as the antibiotic can kill the BCG bacteria and abrogate efficacy [38].

#### Consensus recommendations

The Task Force agreed that oral quinolones (not administered prior to or within 6 h of administering BCG) can reduce toxicity and can be considered for all patients receiving BCG based on Level A evidence.

### What is the role of combination intravesical therapy with interferon-alpha plus BCG?

#### Literature review and analysis

Multiple clinical trials and a meta-analysis have produced conflicting results regarding the success of treatments using BCG with intravesical interferon-alpha versus BCG alone [10–16, 39, 40]. Interferon-alpha has been combined with BCG in several studies and the role of this combination continues to be evaluated. Randomized data among BCG naïve patients suggest similar efficacy of BCG with or without interferon-alpha added [41]. Other reports of the combination are in patients who have recurred after BCG. Some of these results suggest subsequent benefit, but others describe “BCG failure” as a poor prognostic factor for the combination, especially among those deemed truly “BCG unresponsive” [42–44].

#### Consensus recommendations

The Task Force agreed that combination approaches of BCG plus interferon overall seemed generally no more successful than BCG alone based on Level B evidence.

### What are the evaluation criteria following BCG therapy?

#### Literature review and analysis

Prospective trials consistently demonstrate that the timing of recurrence relative to BCG treatments as well as the number of prior courses influences the risk of progression and subsequent response to additional BCG or other treatments. Definitions of failure patterns have been published over the years and are reviewed by the IBCG [24]. Recent discussion regarding the failure pattern have been put forth by Lightfoot et al. [45] and by Kamat et al. for the IBCG, particularly for evaluation in the setting of clinical trials [24]. These include the following:

1. BCG refractory: persistent high-grade disease at 6 months despite adequate BCG treatment. Adequate BCG therapy has been administered when a patient has received at least 5 of 6 doses of induction therapy and at least 1 maintenance (2 of 3 doses) or 1 repeat course (5 of 6 doses). This category also includes any stage/grade progression by 3 months after the first cycle of BCG (i.e., T1 high-grade disease at 3 months).
2. BCG relapsing: recurrence of high-grade disease after achieving a disease-free state at 6 months following adequate BCG (as defined above). For the purpose of being included in the BCG unresponsive category (see below), patients should be within 6–9 months of the last BCG exposure (e.g., patient on maintenance therapy).
3. BCG unresponsive: includes ‘BCG refractory’ and ‘BCG relapsing’ (within 6–9 months of last BCG exposure) patients noted above. This group represents patients for whom further BCG is NOT indicated and radical cystectomy is a true option. Thus, these patients could be considered for single-arm studies, in which they are guaranteed to receive an experimental therapy.
4. BCG resistant (this term is not currently used but is included here for clarity): recurrent or persistent disease 3 months after an induction cycle. In these cases, BCG resistance has resolved 6 months after BCG re-treatment, with or without transurethral resection.

Other recommendations regarding patient evaluation include:

1. Patients who have recurrent disease after adequate BCG should have evaluation of upper tracts and prostatic urethra [46].
2. Patients with increasing disease (number, size, grade, or stage of disease) at the initial 3 month cystoscopic examination should be considered unresponsive to BCG and alternate treatment should be recommended.
  Level of evidence: B
3. Patients with residual or recurrent carcinoma in situ at the 3 month cystoscopy may benefit from 3 additional weekly BCG treatments, but those with disease at 6 months should be considered unresponsive to BCG.
  Level of evidence: B

#### Consensus recommendations

The BCG failure pattern (resistant, refractory, or relapsing) should be considered in making decisions about further therapy.

### Which factors predict response to BCG, and how should response to BCG be monitored?

#### Literature review and analysis

Multiple studies have illustrated that clinical parameters are the strongest predictors of response to intravesical immunotherapy with BCG [10–16]. These parameters include grade, stage, presence of carcinoma in situ, age, and pattern of prior BCG failure. Cystoscopy with cytology at periodic intervals remains the only reliable method to monitor response to BCG [10–16]. However, fluorescence in situ hybridization (FISH) techniques that detect aneuploidy of certain chromosomes in cells voided from the bladder characterizing them as malignant [47] can be used to detect so called molecular recurrence and has been used to risk stratify patients undergoing BCG therapy based on FISH results at early time points [45, 46]. Notably, reflex use of FISH in the setting of suspicious cytology has not yet been shown to modify surveillance strategies [48].

In addition, several groups have developed risk models based on clinical features to help predict response to BCG [49–51], and another recent report evaluated these models and guidelines in patients treated with intravesical chemotherapy [52]. Additional immunologic based assays are being developed, such as the CyPRIT assay, which is a nomogram constructed using urinary levels of cytokines induced by BCG and predicted the likelihood of recurrence with 85.5% accuracy (95% confidence interval: 77.9–93.1%) [53].

#### Consensus recommendations

The Task Force agreed that clinical parameters (grade, stage, and presence of carcinoma in situ) are the strongest predictors of response to intravesical immunotherapy with BCG. While Level B evidence illustrates that urinary FISH monitoring is predictive of response to BCG, the Task Force believes that this remains investigational and should be correlated with clinical evaluation.

### How can patient support during the management of NMIBC enhance access to appropriate management?

#### Literature review and analysis

Approximately 50% of patients with newly diagnosed NMIBC do not receive appropriate therapy with intravesical BCG. Reasons for this are myriad, including reluctance on the part of the patient and the physician, lack of appreciation of the potential benefit, and access to appropriate facilities that can administer BCG. Patient navigation approaches, or support programs developed to help guide patients through the care system, appear to greatly improve the latter, providing timely access to appropriate care [54–56]. Additionally, the Urologic Diseases in America Project has documented the underuse of guideline-recommended care in NMIBC as well as in invasive disease [57–59]. It is proposed that implementing patient navigation programs may reduce the time from diagnosis to treatment of NMIBC and could increase the likelihood of actually undergoing intravesical therapy in eligible survivors. Additionally guideline-appropriate care is likely to improve outcomes for most categories of early bladder cancer. This proposal is extrapolated from a large meta-analysis of patients with abnormal breast, cervical, colorectal, or prostate cancer screening outcomes and the role of patient navigators to facilitate timely cancer care [55].

#### Consensus recommendations

Patient navigation can eliminate barriers to oncologic care, enhance patient decision-making, and improve the patient experience during their cancer care, which his has been demonstrated in screening outcomes for a variety of malignancies. Bladder cancer-specific outcome measures should be developed, validated, and utilized as targets for patient navigation. A formal study of the efficacy of these tools in patients with bladder cancer should be undertaken, particularly given the low rate of compliance with established treatment guidelines.

### What are the most important practical aspects of administration of BCG?

#### Practical issues

##### Literature review and analysis

The use of lidocaine or excessive lubricants during catheterization has been shown to have inhibitory effects on BCG viability. One study in particular, reported significant impairment of BCG viability, dependent on dosage and time of co-incubation, with all lubricants analyzed [60]. Several components of these lubricants, namely lidocaine hydrochloride, glyceryl stearate, propyl-4-hydroxy-benzoate and chlorhexidine digluconate, were identified as responsible for this inhibition. Moreover, the fluid recovered from the bladder after lubricant assisted catheterization also showed an inhibitory effect.

##### Consensus recommendations

**Fig. 2:**
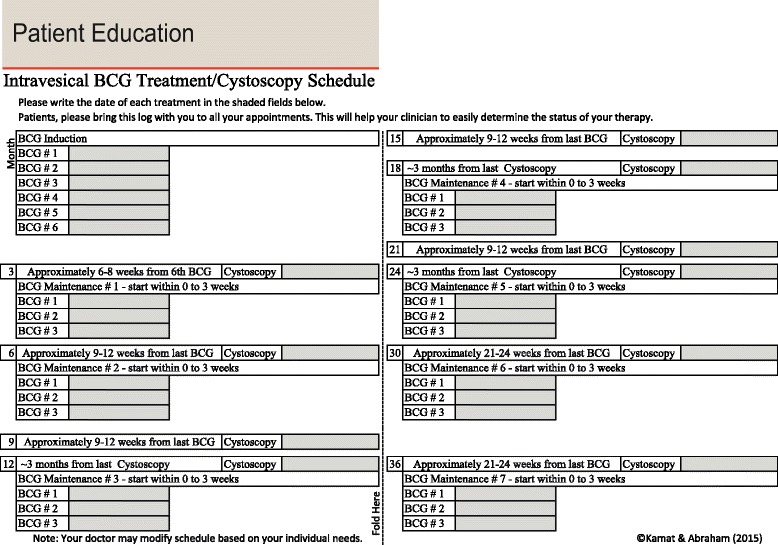
Sample template to provide to patients to record BCG treatment/cystoscopy dates

The use of lidocaine or use of excessive lubricants is not recommended with the administration of intravesical BCG. Additionally, with the use of local anesthetic, patients may not be able to feel/report a potentially traumatic catheterization. When considering other practical issues for BCG administration, the Task Force determined that it is not necessary to rotate patients every 15 min post BCG instillation [38]. Moreover, patients should also be provided with a template that they can use to record BCG treatment/cystoscopy dates (Fig. 2). Patients should bring and complete these with each subsequent visit to the same or other providers.

### Muscle-invasive and metastatic disease

#### What is the current role of immune checkpoint blockade in metastatic urothelial carcinoma?

##### Literature review and analysis

Multiple clinical trials have been undertaken to evaluate the role of immune checkpoint inhibitors in locally advanced and metastatic urothelial carcinoma. IMvigor210 tested treatment with atezolizumab in two cohorts: cisplatin-ineligible patients with locally advanced or metastatic disease (cohort 1) and cisplatin-pre-treated patients with locally advanced or metastatic disease (cohort 2) [61–63]. Cohort 1 enrolled patients with metastatic disease who were chemotherapy-naïve and cisplatin ineligible. The overall response rate (ORR) in this cohort was 23% (27/119), and responses occurred in all subgroups regardless of PD-L1 expression. At the time of reporting, the median duration of response was not reached. Median survival was 15.9 months among all patients [61]. Cohort 2 enrolled patients who had received at least one prior line of platinum chemotherapy; 41% had received at least two. The objective response proportion in cohort 2 was 15% and was greater (26%) in patients with high expression of PD-L1 on immune cells (IC2/3) [62, 63]. Overall, 84% of responses were ongoing at a median of 11.7 months, and the median duration of response had not been reached. The median progression-free survival was short in all subgroups (2.1 months). However, the median overall survival was 11.4 months in the high PD-L1 group (IC2/3), and 7.9 months in the overall cohort [62, 63]. Based on these results, the FDA granted atezolizumab accelerated approval for use in patients with locally advanced or metastatic urothelial carcinoma who have disease progression during or following treatment with platinum-based chemotherapy for metastatic disease, or who have disease progression within 1 year of neoadjuvant or adjuvant treatment with platinum-containing chemotherapy. Based on data from cohort 1 of the IMvigor210 trial, atezolizumab was subsequently granted accelerated approval for frontline treatment of patients with locally advanced or metastatic urothelial carcinoma who are ineligible for cisplatin chemotherapy [64]. For all approvals granted under the accelerated approval pathway based on response rate and duration of response, continued approval may be contingent on evidence of clinical benefit in further trials.

Phase I/II data from the CheckMate 032 study have also been reported for nivolumab, a PD-1 inhibitor. In this study, 78 patients who previously received platinum-based chemotherapy were treated with single agent nivolumab. Objective responses were observed in 24.4% of patients and median overall survival in this study was 9.7 months [65]. Checkmate 275, a single arm phase II study of nivolumab as a single agent, enrolled 270 patients with locally advanced or metastatic urothelial carcinoma who had progressed following platinum-based chemotherapy [66]. Objective responses were observed in 19.6% of patients. Higher levels of PD-L1 expression on tumor cells were associated with higher objective response rates (28.4% with PD-L1 expression >5%; 23.8% with PD-L1 expression ≥1%; and 16.1% with PD-L1 expression <1%). Based on these results, the FDA granted nivolumab accelerated approval for use in patients with locally advanced or metastatic urothelial carcinoma who have disease progression during or following treatment with platinum-based chemotherapy for metastatic disease, or who have disease progression within 1 year of neoadjuvant or adjuvant treatment with platinum-containing chemotherapy.

Durvalumab, a PD-L1 inhibitor, was tested in 61 patients with previously treated metastatic urothelial carcinoma [67]. In this study, the first 20 patients were enrolled regardless of PD-L1 status; however, the remainder were required to have ≥5% of tumor cells expressing PD-L1. In 42 evaluable patients, the objective response rate was 31%; in patients whose tumors stained positive for PD-L1 (≥25% of tumor or tumor-infiltrating immune cells), the objective response rate was 46% compared with 0% in patients whose tumors were PD-L1 negative. As a result of these, and more recent data, durvalumab received accelerated FDA approval in May 2017 for the treatment of patients with locally advanced or metastatic urothelial carcinoma who have disease progression during or after platinum-containing chemotherapy, or within 12 months of neoadjuvant or adjuvant treatment with platinum-containing chemotherapy. (See Additional file 1 for comments and as yet unpublished data on durvalumab).

A second PD-L1 inhibitor, avelumab, was evaluated in patients with locally advanced or metastatic urothelial carcinoma that failed to respond to platinum-based therapy, as part of the JAVELIN Solid Tumor Trial (NCT01772004). Based on data available at the time [confirmed ORR = 13.3% and 16.1% at minimum follow-up of 13 weeks (*n* = 226) and 6 months (*n* = 161), respectively; median duration of response not reached in either group; no difference in response ate based on PD-L1 tumor expression in the 84% of patients who were evaluable] avelumab received accelerated approval for patients with locally advanced or metastatic urothelial carcinoma whose disease progressed during or following platinum-containing chemotherapy, or within 12 months of neoadjuvant or adjuvant platinum-containing chemotherapy [68]. Subsequently published clinical data have confirmed the efficacy of avelumab in this indication [69].

Data were also recently presented from the Keynote-045 phase III study of pembrolizumab, an anti-PD-1 antibody, vs. investigator’s choice of chemotherapy (paclitaxel, docetaxel, or vinflunine) [70, 71]. The study was stopped early based on a pre-specified interim analysis in which pembrolizumab demonstrated a significant improvement in overall survival (median 10.3 vs. 7.4, HR: .73, *p* = .0022). The Keynote-045 full report shows pembrolizumab to be the first therapy to demonstrate a significant survival advantage over chemotherapy [71]; as of June 2017, pembrolizumab remains the only agent showing such an advantage in a phase III trial. Moreover, the open-label, phase II Keynote-052 study demonstrated an objective response rate of 24% in 100 treatment-naïve, cisplatin ineligible patients treated with pembrolizumab [72]. Based on these two trials, pembrolizumab was granted two separate approvals in urothelial cancer: regular approval as second line therapy for patients whose disease has progressed with platinum-containing chemotherapy, or within 12 months of neoadjuvant or adjuvant treatment with platinum-containing chemotherapy, and accelerated approval as frontline therapy in cisplatin-ineligible patients [73].

In the first approval of its kind, the FDA recently granted accelerated approval for use of pembrolizumab in solid tumors demonstrated to be microsatellite instability-high (MSI-H) or mismatch repair-deficient (dMMR), in patients with disease progression after prior treatment and who have no satisfactory alternative treatment options. This is the first FDA approval based on the presence of a tumor biomarker as opposed to tumor site and, as such, broadens treatment options for a subset of patients with a variety of malignancies, including urothelial carcinoma. The approval was based on data from 149 patients enrolled in five uncontrolled, single-arm clinical trials across 15 cancer types, of whom 39.6% achieved complete or partial response. Within this responder group, 78% of patients had response lasting ≥6 months [73].

In the non-randomized CheckMate 032 study, preliminary data have also been reported on the combination of nivolumab plus ipilimumab in metastatic urothelial carcinoma. At time of presentation the nivolumab (1 mg) combined with ipilimumab (3 mg) group had an overall response rate of 38.5%, while the nivolumab (3 mg) combined with ipilimumab (1 mg) and nivolumab monotherapy groups had overall response rates of 26% and 25.5%, respectively [70]. Overall, these results indicate that targeting the immune system shows significant promise for the treatment of metastatic urothelial carcinoma.

##### Consensus recommendations

**Fig. 3:**
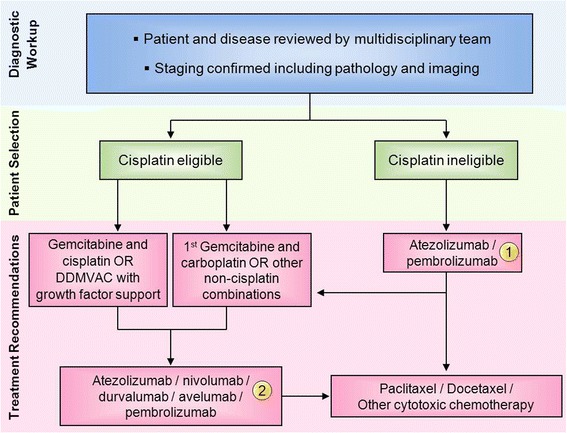
All of the treatment options shown may be appropriate. The selection of therapy should be individualized based on patient eligibility and the availability of therapy, at the discretion of the treating physician. These algorithms represent the consensus recommendations of the Task Force. (1) Atezolizumab and pembrolizumab are FDA approved for patients with metastatic urothelial carcinoma who are ineligible to receive cisplatin. (2) Atezolizumab, nivolumab, durvalumab, avelumab, and pembrolizumab are FDA approved for advanced disease that has worsened on platinum containing regimens or within 12 months of receiving a platinum-containing regimen before (neoadjuvant) or after surgery (adjuvant). Abbreviations: dose-dense methotrexate, vinblastine, doxorubicin, and cisplatin (DDMVAC)

Atezolizumab, durvalumab, avelumab, pembrolizumab and nivolumab are all currently FDA-approved and recommended for treatment of patients with locally advanced or metastatic urothelial carcinoma previously treated with platinum-based chemotherapy or relapsed within 12 months of perioperative platinum-based chemotherapy. Pembrolizumab demonstrated improved survival and is the only agent with Level A evidence at this time. There are currently no evident reasons to select one agent over the others, other than the practical matters of dosing and convenience. Atezolizumab and pembrolizumab are also recommended as first-line theraphy in cisplastin-ineligible patients (Fig. 3). Finally, pembrolizumab is an appropriate choice of treatment in any patient whose tumor has the MSI-H biomarker and whose disease has progressed following prior treatment, with no satisfactory alternative treatment options.

#### Should PD-L1 staining be used routinely in clinical practice?

PD-L1 staining using the Ventana SP142 assay (atezolizumab) or SP263 assay (durvalumab) appears to identify a patient population more likely to respond to anti-PD-L1 therapy in the chemotherapy-refractory setting. However, in both cases durable responses were observed in patients even with low levels of PD-L1 expression, albeit at lower frequencies. PD-L1 has been shown to be a potentially dynamic biomarker, and the relevance of archival tumor to the current immune status of the tumor is unclear. Other PD-L1 assays are available, but none have been validated as a diagnostic in urothelial carcinoma.7

##### Consensus recommendations

Currently, the data do not support using PD-L1 immunohistochemistry to select patients for treatment. However, the FDA has approved complementary assays for evaluating PD-L1 expression when considering treatment with atezolizumab (Ventana PD-L1 SP142) and durvalumab (Ventana PD-L1 SP263) in urothelial carcinoma. This will lead to ongoing evaluation of this aspect of patient selection.

## Future directions

### Ongoing development of novel and/or systemic immunotherapy in NMIBC, muscle-invasive bladder cancer, and metastatic bladder cancer

#### What criteria should be considered for the development of systemic immunotherapies for treatment of NMIBC?

The development of systemic immunotherapies for treatment of NMIBC should be considered if they offer a mechanistic advantage or pharmacokinetic advantage to intravesical therapy. Furthermore, such administration could be considered for practical reasons if intravesical therapy of the experimental agent is not deemed feasible.

Clinical investigation of systemic treatments for NMIBC should be based on the following considerations:

- Mechanism of action of the intervention
- Feasibility of clinical investigation
- Potential systemic toxicities in the context of the natural/treated history of the underlying disease state
- Pharmacology demonstrating adequate bladder exposure when administered systemically, or the drug doesn’t require direct contact with tumor cells.

High risk NMIBC is particularly well-suited for clinical investigation based on these considerations. Appropriate clinical trial design in NMIBC is essential to provide the most clinically relevant data for each specific disease-risk category of interest. Recently, the IBCG developed formal recommendations regarding key definitions, end points, and overall clinical trial design for NMIBC to encourage uniformity and promote the development of new agents in this disease setting [24]. Highlights from these recommendations include the need to develop eligibility criteria and evaluations on the disease risk category as well as to properly record the type of failure for BCG (unresponsive, refractory, relapsed, or intolerant). In general, the IBCG recommends using time-to-recurrence or recurrence-free survival as a primary end point, while time to progression, toxicity, disease-specific survival, and overall survival as secondary end points [24]. A list of selected ongoing immunotheraphy trials in bladder cancer is provided in Table 1. 

##### Recommendations for future development

The Task Force discussed several issues and areas of further investigation that should be addressed in future recommendations:

1. Clinical trials of novel immunotherapy in both muscle-invasive and metastatic bladder cancer should explore the potential role for integral biomarkers for the selection of patients most likely to benefit.
2. Clinical trials of novel immunotherapy in both muscle-invasive and metastatic bladder cancer should explore the potential role for a genetic basis for response including exome analysis and intrinsic bladder cancer subtypes.
3. T cell infiltration is an important prognostic finding in urothelial cancer, but this measurement may be confounded by dynamic changes (i.e., interaction with therapy). This requires further evaluation and validation before a recommendation can be made.
4. Selection of patients for clinical trials of systemic immune therapies based on tissue expression of a single immune biomarker with measurement via immunohistochemistry is currently not justified in the post-platinum population. However, investigation of chemotherapy-sparing regimens in the first line setting remains an important area of research.
5. Biomarker development for immunotherapy agents may require integration of multiple biologic components as opposed to a single marker.
6. Immune checkpoint inhibitor strategies should be investigated across disease states of urothelial carcinoma, though toxicity may limit use in certain disease states. Combination approaches using immune checkpoint blockade are also warranted.
7. A formal study of patient navigation tools in patients with early and locally advanced disease is warranted.

## Additional files

Additional file 1: Comments from Open Review. Comments received during open review of this consensus statement (PDF 94 kb)

Additional file 2: Cancer Immunotherapy Guidelines-Bladder Task Force Roster. The full listing of the Task Force roster (DOCX 12 kb)

Additional file 3: Pre-Meeting Survey Questions and Responses. The pre-meeting survey questions and answers for the Task Force meeting (Task Force Pre-Meeting Survey Questions and Answers) (DOCX 21 kb)

Additional file 4: Cancer Immunotherapy Guidelines-Bladder Bibliography. The full bibliography (Cancer Immunotherapy Guidelines-Bladder Cancer). The literature search was performed as outlined in the Methods section of this manuscript (PDF 215 kb)

## Abbreviations

AUA: American Urologic Association
BCG: Bacillus Calmette-Guérin
dMMR: mismatch repair-deficient
EAU: European Association of Urology
FDA: U.S. Food and Drug Administration
IBCG: International Bladder Cancer Group
ICUD: International Consultation on Urological Diseases
MSI-H: Microsatellite instability-high
NAM: National Academy of Medicine
NCCN: National Comprehensive Cancer Network
NMIBC: non-muscle invasive bladder cancer
SITC: Society for Immunotherapy of Cancer
