# Erratum to: Society for Immunotherapy of Cancer consensus statement on immunotherapy for the treatment of bladder carcinoma

**DOI:** 10.1186/s40425-017-0280-z

**Published:** 2017-09-28

**Authors:** Ashish M. Kamat, Joaquim Bellmunt, Matthew D. Galsky, Badrinath R. Konety, Donald L. Lamm, David Langham, Cheryl T. Lee, Matthew I. Milowsky, Michael A. O’Donnell, Peter H. O’Donnell, Daniel P. Petrylak, Padmanee Sharma, Eila C. Skinner, Guru Sonpavde, John A. Taylor, Prasanth Abraham, Jonathan E. Rosenberg

**Affiliations:** 1 University of Texas MD Anderson Cancer Center, 1515 Pressler Unit 1373, Houston, TX 77030 USA; 2 Dana-Farber Cancer Institute, Brookline, MA 02446 USA; 3 Tisch Cancer Institute at Mount Sinai Medical Center, New York, NY 10029 USA; 4 University of Minnesota, Minneapolis, MN 55455 USA; 5 BCG Oncology, Phoenix, AZ 85032 USA; 6 Bladder Cancer Advocacy Network, North Carolina Triangle Chapter, Chapel Hill, NC 27517 USA; 7 The Ohio State University Wexner Medical Center, Columbus, OH 43210 USA; 8 University of North Carolina, Chapel Hill, NC 27599 USA; 9 University of Iowa, Iowa City, IA 52242 USA; 10 University of Chicago, Chicago, IL 60637 USA; 11 Yale Cancer Center, New Haven, CT 06520 USA; 12 University of Texas MD Anderson Cancer Center, Houston, TX 77030 USA; 13 Stanford University, Stanford, CA 94305 USA; 14 University of Alabama, Birmingham, AL 35294 USA; 15 University of Kansas Cancer Center, Kansas City, KS 66160 USA; 16 University of Texas MD Anderson Cancer Center, Houston, TX 77030 USA; 17 Memorial Sloan Kettering Cancer Center, New York, NY 10065 USA

## Erratum

After the publication of the article [1], the treatment algorithm for advanced/metastatic bladder cancer in Fig. 3 was updated to reflect the current use of immunotherapy in this setting. The correct Fig. 3 can be seen here and the original article has been updated to reflect this change.

**Fig. 3:**
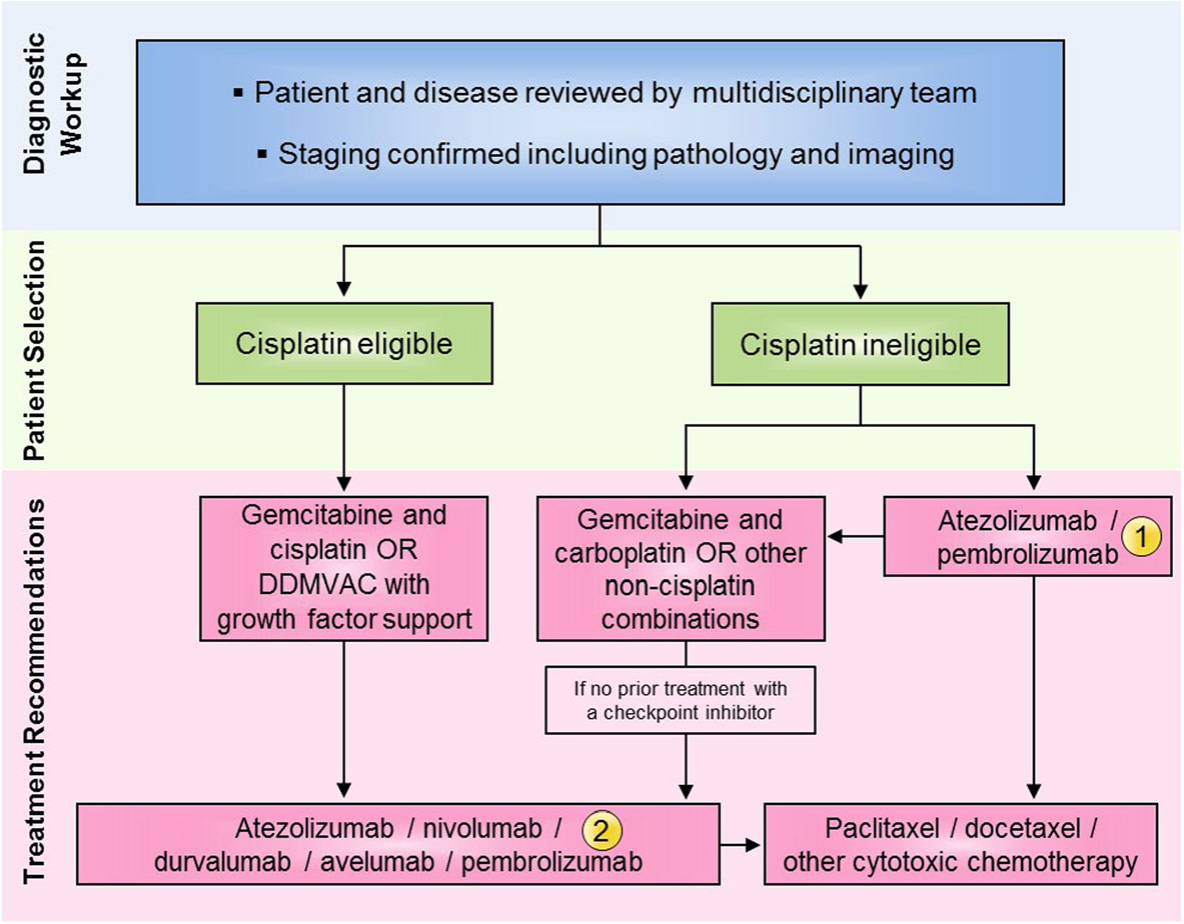
All of the treatment options shown may be appropriate. The selection of therapy should be individualized based on patient eligibility and the availability of therapy, at the discretion of the treating physician. These algorithms represent the consensus recommendations of the Task Force. (1) Atezolizumab and pembrolizumab are FDA approved for patients with metastatic urothelial carcinoma who are ineligible to receive cisplatin. (2) Atezolizumab, nivolumab, durvalumab, avelumab, and pembrolizumab are FDA approved for advanced disease that has worsened on platinum containing regimens or within 12 months of receiving a platinum-containing regimen before (neoadjuvant) or after surgery (adjuvant). Abbreviations: dose-dense methotrexate, vinblastine, doxorubicin, and cisplatin (DDMVAC)
