# Supplementary material for: Society for Immunotherapy of Cancer consensus statement on immunotherapy for the treatment of bladder carcinoma
Source: J Immunother Cancer. 2017 Aug 15;5:68. doi: 10.1186/s40425-017-0271-0 (PMC5557323; doi:10.1186/s40425-017-0271-0)
Supplement: Supplementary file 1 — Comments from Open Review. Comments received during open review of this consensus statement (PDF 94 kb) [file 40425_2017_271_MOESM1_ESM.pdf]

AstraZeneca Comments on Society for Immunotherapy of Cancer Consensus Statement on Immunotherapy for the Treatment of Bladder Carcinoma

Following are comments from AstraZeneca on the Society for Immunotherapy of Cancer Consensus Statement on Immunotherapy for the Treatment of Bladder Carcinoma ("Consensus Statement"). AstraZeneca is commenting on the Consensus Statement in order to provide more current information regarding IMFINZI and the comments below should in no way be construed as a recommendation for its use.

Comment Number	Pg.	Original Text	Comments or Proposed Replacement Text (Suggested Edits in Red)	Rationale for Change
**1**	15	First paragraph, third line: Durvalumab, a PD-L1 inhibitor, was tested in 61 patients with previously treated metastatic urothelial carcinoma. In this study, the first 20 patients were enrolled regardless of PD-L1 status; however, the remaining were required to have ≥5% of tumor cells expressing PD-L1. In 42 response evaluable patients, the objective response rate was 31%; in patients whose tumors stained positive for PD-L1 (≥25% of tumor or tumor-infiltrating immune cells), the objective response rate was 46% and 0% in patients whose tumors were PD-L1 negative. As a result, durvalumab has been given breakthrough status by the FDA for second-line treatment of patients with metastatic urothelial carcinoma.	Replace with: IMFINZI™ (durvalumab), a PD-L1 blocking antibody, was studied in patients with locally advanced or metastatic urothelial carcinoma. In the 94 post-platinum patients (≥2L), median overall survival was 14.1 months, progression-free survival was 2.2 months, and disease control rate was 42.6%[1]. The approval of durvalumab is based on safety and efficacy in 182 patients with additional follow up from the same study[2]. In the overall population (N=182), objective response rate (ORR) was 17.0%; in patients categorized as PD-L1 high (N=95, defined as ≥25% of tumor cell staining or immune cell staining), ORR was 26.3%, and in those who were PD-L1 low/negative (N=73, defined as <25% of tumor cell and immune cell staining), ORR was found to be 4.1%[2]. Among the 31 responding patients, 14 (45%) had ongoing responses of ≥6 months and 5 patients (16%) had ongoing responses of ≥12 months[2]. Durvalumab was approved by the US FDA in May 2017 for the treatment of patients with locally advanced or metastatic urothelial carcinoma who have disease progression during or following platinum-containing chemotherapy, or have disease progression within 12 months of neoadjuvant or	The data described in this section are from an earlier data cut, which has been updated with the data from the IMFINZI™ (durvalumab) Prescribing Information[2]. Therefore, we suggest the replacement to reflect the most current and complete data available on durvalumab. The first 20 patients were enrolled regardless of PD-L1 expression; however, preliminary data suggested that PD-L1 may be expressed more commonly on ICs than on TCs. Therefore, to ensure assessment of the contribution of PD-L1–expressing TCs to response with durvalumab, subsequent patients were required to have ≥5% PD-L1 expression on TCs, until a protocol amendment removed this requirement[3]. Forty-three patients were enrolled with this requirement prior to the amendment[3]. Although it might be expected that this could result in selection bias, an interim analysis of the primary efficacy population showed that ORR in patients with <5% PD-L1 expression on TCs was similar to ORR in the overall population[3].

			adjuvant treatment with platinum-containing chemotherapy[2]. This indication is approved under accelerated approval based on tumor response rate and duration of response[2]. Continued approval for this indication may be contingent upon verification and description of clinical benefit in confirmatory trials[2].	
**2**	15	Second paragraph, first line: Atezolizumab and nivolumab are currently the only FDA approved therapies for locally advanced or metastatic patients with disease progression on platinum-based chemotherapy.	Replace with: Atezolizumab, nivolumab and durvalumab are currently FDA approved therapies for locally advanced or metastatic patients with disease progression on platinum-based chemotherapy.	The statement should accurately reflect the regulatory approval of durvalumab.
**3**	15	Second paragraph, third line: Currently, both atezolizumab and nivolumab are recommended as treatment for patients with locally advanced metastatic urothelial carcinoma previously treated with platinum-based chemotherapy or relapsed within 12 months of perioperative platinum based chemotherapy.	Replace with: Currently, atezolizumab, durvalumab, and nivolumab are recommended as treatment for patients with locally advanced metastatic urothelial carcinoma previously treated with platinum-based chemotherapy or relapsed within 12 months of perioperative platinum based chemotherapy.	The statement should accurately reflect the FDA approved indication for durvalumab. Durvalumab is indicated for the treatment of patients with locally advanced or metastatic urothelial carcinoma who have disease progression during or following platinum-containing chemotherapy, or have disease progression within 12 months of neoadjuvant or adjuvant treatment with platinum-containing chemotherapy[2]. This indication is approved under accelerated approval based on tumor response rate and duration of response[2]. Continued approval for this indication may be contingent upon verification and description of clinical benefit in confirmatory trials[2].
**4**	15	Second paragraph, eighth line: Durvalumab and avelumab, both anti-PD-L1 antibodies, are also being evaluated for accelerated approval by the US FDA.	Remove durvalumab from the statement: For example: “Avelumab,… is also being evaluated…”	Durvalumab is currently approved by the FDA, as outlined above. AstraZeneca cannot comment on the regulatory status of avelumab.
**5**	16	First paragraph, first line:	Add statement: Staining for PD-L1 expression using the Ventana	As indicated above, in the patients identified as PD-L1 high, ORR was

		PD-L1 staining using the Ventana SP142 assay appears to identify a patient population more likely to respond to anti-PD-L1 therapy with atezolizumab in the chemotherapy-refractory setting.	SP263 assay also appears to identify patients more likely to respond to durvalumab[2].	26.3%, while in patients with PD-L1 low/negative status, ORR was 4.1%[2].
**6**	16	First paragraph, third line: However, durable responses were observed in patients even with low levels of PD-L1 expression (IC0/1), albeit at lower frequencies.	Add statement: In the durvalumab clinical trial using Ventana SP263, durable responses were also observed in the PD-L1 low/negative group[2].	As presented in the durvalumab Prescribing Information, durable responses were observed not only in the overall and PD-L1 high populations (duration of response was not reached for both), but also in the PD-L1 low/negative group (12.3 months); of five patients who had complete response, one patient had PD-L1 low/negative status[2].
**7**	16	First paragraph, fifth line: Other PD-L1 antibodies are available, but none have been validated with the currently available FDA-approved agents, atezolizumab and nivolumab.	Add statement: Ventana PD-L1 (SP263) assay has been clinically validated for use with durvalumab.	PD-L1 high status, as determined by Ventana PD-L1 (SP263) assay, is associated with increased ORR in a single-arm study of durvalumab[4]. AstraZeneca cannot comment on atezolizumab and nivolumab.
**8**	16	Second paragraph, second line: The FDA has approved a complementary assay (Ventana SP142) for evaluating PD-L1 expression when considering treatment with atezolizumab in urothelial carcinoma, and this will lead to ongoing evaluation of this parameter.	Add statement: The FDA has also approved a complementary diagnostic (Ventana PD-L1 SP263) to identify PD-L1 expression levels in patients considering treatment with durvalumab in urothelial carcinoma.	Ventana PD-L1 (SP263) Assay is a qualitative immunohistochemical assay using rabbit monoclonal anti-PD-L1 clone SP263 intended for use in the assessment of the PD-L1 protein in formalin-fixed, paraffin-embedded (FFPE) urothelial carcinoma tissue stained with OptiView DAB IHC Detection Kit on a Ventana BenchMark ULTRA instrument[4]. PD-L1 high status, as determined by Ventana PD-L1 (SP263) assay, is associated with increased ORR in a single-arm study of durvalumab[4].

**9**	17	Bullet 4:<br>Selection of patients for clinical trials of systemic immune therapies based on tissue expression of a single immune biomarker with measurement via immunohistochemistry is currently not justified.	Replace with:<br>Selection of patients for clinical trials of systemic immune therapies based on tissue expression of a single immune biomarker with measurement via immunohistochemistry is currently not justified in the post-platinum population. However, it remains an important area of research to investigate chemotherapy-sparing regimens in the first line setting.	Based on the current paucity of data regarding the utility of immune biomarkers to select patients for clinical trials in the first line setting, it is AstraZeneca's position that the use of an immune biomarker in future clinical trials of first line approaches studying chemotherapy-sparing regimens remains a promising area of research.
**10**	28	Refractory Patients box (orange):<br>Atezolizumab/nivolumab	Add:<br>Durvalumab, so that it reads:<br>atezolizumab/durvalumab/nivolumab	Durvalumab is indicated for the treatment of patients with locally advanced or metastatic urothelial carcinoma who have disease progression during or following platinum-containing chemotherapy, or have disease progression within 12 months of neoadjuvant or adjuvant treatment with platinum-containing chemotherapy[2].
**11**	29	Table 1, row 8, middle column:<br>Phase II vs. standard of care chemotherapy	Replace with:<br>Phase III vs. standard of care chemotherapy[5]	The table should reflect the correct trial phase (NCT02516241).
**12**	30	Table 1, row 1	Delete	This information is redundant with above (same NCT number).
**13**	29-30	Table 1	Suggest adding the following information:<br>• Biomarker-directed multi-drug study, NCT02546661; Phase 1b; muscle invasive bladder cancer[6]<br>• Durvalumab in Combination with Tremelimumab, NCT02261220; Phase 1; advanced solid tumors including urothelial carcinoma[7]	This change could be implemented to provide more comprehensive information regarding the durvalumab clinical trial program in bladder cancer.

References:

1. Powles T, O'Donnell PH, Massard C, et al. Updated efficacy and tolerability of durvalumab in locally advanced or metastatic urothelial carcinoma [poster]. Presented at: American Society of Clinical Oncology – Genitourinary Cancers Symposium; February 16-18, 2017; Orlando, FL.
2. IMFINZI Prescribing Information.
3. In house data. AstraZeneca Pharmaceuticals LP. Second Interim Clinical Study Report CD-ON-MEDI4736-1108 (v1.0).
4. Ventana PD-L1 (SP263) Assay. Package Insert. Ventana Medical Systems, Inc. May 2017.
5. National Institutes of Health. Study of MEDI4736 with or without tremelimumab versus standard of care chemotherapy in bladder cancer. ClinicalTrials.gov web site. https://clinicaltrials.gov/ct2/show/NCT02516241. Accessed May 16, 2017.
6. National Institutes of Health. Open-Label, Randomised, Multi-Drug, Biomarker-Directed, Phase 1b Study in Pts w/ Muscle Invasive Bladder Cancer (BISCAY). ClinicalTrials.gov website. https://clinicaltrials.gov/ct2/show/NCT02546661. Accessed May 16, 2017.
7. National Institutes of Health. Study of MEDI4736 in Combination With Tremelimumab in Subjects With Advanced Solid Tumors. ClinicalTrials.gov website. https://clinicaltrials.gov/ct2/results?term=NCT02261220. Accessed May 16, 2017.

Dear SITC Members,

Thank you for the opportunity to provide comments on the "guidelines for bladder cancer". I have reviewed this document, and have the following 2 specific recommendations.

1. In section 'literature review and analysis" – in pages 14 and 15, please add data from the following information, all of which is public.

The FDA has accepted avelumab for accelerated review in urothelial cancer in early 2017. Data for avelumab in bladder cancer was presented by Patel et al at ASCO GU 2017. It was also published in JCO by Andrea Apolo et al. as a Rapid report. The link to the published manuscript is here.

Avelumab, an Anti–Programmed Death-Ligand 1 Antibody, In Patients With Refractory Metastatic Urothelial Carcinoma: Results From a Multicenter, Phase Ib Study.

2. Table 1 on page 29 under the column "drug / agent" - the name of the drug should be changed to "avelumab". Not "maintenance avelumab".

Looking forward to the final version of the consensus guidelines in print.

Andy Blake-Haskins PharmD
Immuno-oncology Asset Team Leader
Pfizer
San Diego CA
