# Supplementary material for: Society for Immunotherapy of Cancer consensus statement on immunotherapy for the treatment of bladder carcinoma
Source: J Immunother Cancer. 2017 Aug 15;5:68. doi: 10.1186/s40425-017-0271-0 (PMC5557323; doi:10.1186/s40425-017-0271-0)
Supplement: Supplementary file 2 — Cancer Immunotherapy Guidelines-Bladder Task Force Roster. The full listing of the Task Force roster (DOCX 12 kb) [file 40425_2017_271_MOESM2_ESM.docx]

**ADDITIONAL FILE I: Cancer Immunotherapy Guidelines-Bladder Task Force Roster**

**Steering Committee:**

Ashish M. Kamat, MD, MBBS, FACS University of Texas MD Anderson Cancer Center

Jonathan E. Rosenberg, MD Memorial Sloan Kettering Cancer Center

**Task Force Participants:**

Prasanth Abraham, RN University of Texas MD Anderson Cancer Center

Joaquim Bellmunt, MD Dana-Farber Cancer Institute

Matthew D. Galsky, MD Tisch Cancer Institute at Mount Sinai Medical Center

Badrinath R. Konety, MD, MBA University of Minnesota

Donald L. Lamm, MD, FACS Bladder Cancer Genitourinary Oncology

David Langham Bladder Cancer Advocacy Network North Carolina Triangle Chapter

Cheryl Lee, MD The Ohio State University

Matthew I. Milowsky, MD University of North Carolina

Michael A. O’Donnell, MD University of Iowa

Peter H. O’Donnell, MD University of Chicago

Daniel P. Petrylak, MD Yale Cancer Center

Padmanee Sharma, MD, PhD University of Texas M.D. Anderson Cancer Center

Eila C. Skinner, MD Stanford University

Guru Sonpavde, MD University of Alabama

John A. Taylor III, MD University of Kansas
