# Supplementary material for: Society for Immunotherapy of Cancer consensus statement on immunotherapy for the treatment of bladder carcinoma
Source: J Immunother Cancer. 2017 Aug 15;5:68. doi: 10.1186/s40425-017-0271-0 (PMC5557323; doi:10.1186/s40425-017-0271-0)
Supplement: Supplementary file 3 — Pre-Meeting Survey Questions and Responses. The pre-meeting survey questions and answers for the Task Force meeting (Task Force Pre-Meeting Survey Questions and Answers) (DOCX 21 kb) [file 40425_2017_271_MOESM3_ESM.docx]

| 1. **What best describes your primary role in bladder cancer?:** | |
| --- | --- |
| **Answer Options** | **Response Percent** |
| Medical Oncologist | 46.2% |
| Surgical Oncologist | 30.8% |
| Radiation Oncologist | 0.0% |
| Nurse | 15.4% |
| Patient or Patient Advocate | 7.7% |
| Other (please specify) | 0.0% |
| 1. **Which of the following is the primary focus of your clinical activity?:** | |
| **Answer Options** | **Response Percent** |
| Management of non-muscle invasive bladder cancer | 7.7% |
| Management of muscle invasive and locally advanced bladder cancer | 7.7% |
| Management of recurrent/metastatic bladder cancer | 7.7% |
| A and B | 23.1% |
| B and C | 38.5% |
| A and C | 7.7% |
| All of the above | 7.7% |
| None/Not applicable | 0.0% |
| Other (please specify) | 0.0% |
| 1. **With which of the following do you have clinical experience? (select all that apply):** | |
| **Answer Options** | **Response Percent** |
| Chemotherapy | 69.2% |
| Immunotherapy | 100.0% |
| Surgery | 53.8% |
| Targeted therapies | 53.8% |
| Clinical Trials | 76.9% |
| Not applicable | 0.0% |
| 1. **For patients with NMIBC, do you treat variant histology the same as conventional Urothelial carcinoma?** | |
| **Answer Options** | **Response Percent** |
| Yes | 7.7% |
| No | 53.8% |
| Not applicable | 38.5% |
| 1. **For patients with NMIBC, stage TaLG, which of the following do use or recommend?:** | |
| **Answer Options** | **Response Percent** |
| TURBT alone | 7.7% |
| TURBT plus single dose chemotherapy | 53.8% |
| TURBT plus adjuvant intravesical chemotherapy | 0.0% |
| TURBT plus induction BCG immunotherapy | 0.0% |
| TURBT plus induction BCG immunotherapy and maintenance therapy | 7.7% |
| Radical Cystectomy | 0.0% |
| Radiation Therapy | 0.0% |
| Not applicable | 30.8% |
| 1. **For patients with NMIBC, stage TaHG, which of the following do use or recommend?:** | |
| **Answer Options** | **Response Percent** |
| TURBT alone | 0.0% |
| TURBT plus single dose chemotherapy | 7.7% |
| TURBT plus adjuvant intravesical chemotherapy | 0.0% |
| TURBT plus induction BCG immunotherapy | 23.1% |
| TURBT plus induction BCG immunotherapy and maintenance therapy | 46.2% |
| Radical Cystectomy | 0.0% |
| Radiation Therapy | 0.0% |
| Not applicable | 23.1% |
| 1. **For patients with NMIBC, stage T1HG, which of the following do use or recommend?:** | |
| **Answer Options** | **Response Percent** |
| TURBT alone | 7.7% |
| TURBT plus single dose chemotherapy | 0.0% |
| TURBT plus adjuvant intravesical chemotherapy | 0.0% |
| TURBT plus induction BCG immunotherapy | 0.0% |
| TURBT plus induction BCG immunotherapy and maintenance therapy | 46.2% |
| Radical Cystectomy | 15.4% |
| Radiation Therapy | 0.0% |
| Not applicable | 30.8% |
| 1. **For patients with NMIBC, CIS, which of the following do use or recommend?:** | |
| **Answer Options** | **Response Percent** |
| TURBT alone | 0.0% |
| TURBT plus single dose chemotherapy | 7.7% |
| TURBT plus adjuvant intravesical chemotherapy | 0.0% |
| TURBT plus induction BCG immunotherapy | 0.0% |
| TURBT plus induction BCG immunotherapy and maintenance therapy | 53.8% |
| Radical Cystectomy | 7.7% |
| Radiation Therapy | 0.0% |
| Not applicable | 30.8% |
| 1. **Do you believe that papillary tumors and CIS can be grouped together for considerations of therapy and clinical trials?** | |
| **Answer Options** | **Response Percent** |
| Yes | 0.0% |
| Yes, if papillary tumors are high grade | 38.5% |
| No | 30.8% |
| No opinion | 30.8% |
| 1. **What time point do you use to determine failure of immunotherapy?:** | |
| **Answer Options** | **Response Percent** |
| 3 months for all tumors (after induction BCG) | 0.0% |
| 3 months for papillary tumors (after induction BCG) and 6 months for CIS (after induction BCG and maintenance) | 30.8% |
| 6 months for all tumors (after induction BCG and maintenance) | 38.5% |
| Not applicable | 30.8% |
| 1. **For patients who have failed BCG after induction and at least one maintenance course, what is the best comparator arm?:** | |
| **Answer Options** | **Response Percent** |
| None other than radical cystectomy | 53.8% |
| Placebo | 15.4% |
| Intravesical chemotherapy | 15.4% |
| More BCG | 15.4% |
| 1. **Besides immunotherapy with BCG, what other agents do you have experience with or have you used? (select all that apply):** | |
| **Answer Options** | **Response Percent** |
| Interferon | 7.7% |
| KLH | 0.0% |
| Bropiramine | 7.7% |
| Genetically modified BCG | 0.0% |
| Adenoviral agents | 15.4% |
| Checkpoint immune inhibitors | 23.1% |
| None | 23.1% |
| Other (please specify) | 23.1% |
| 1. **Do you feel that systemic immunotherapy is appropriate to test in front-line BCG-eligible patients as monotherapy (i.e. Instead of BCG)?** | |
| **Answer Options** | **Response Percent** |
| Yes | 38.5% |
| No | 46.2% |
| No opinion | 15.4% |
| 1. **For muscle invasive cisplatin-eligible patients, which of the following regimens do you commonly use or recommend as neoadjuvant therapy for muscle invasive bladder cancer? (select all that apply):** | |
| **Answer Options** | **Response Percent** |
| Gemcitabine/cisplatin | 69.2% |
| Dose dense gemcitabine/cisplatin | 23.1% |
| M-VAC | 23.1% |
| Dose dense M-VAC | 53.8% |
| Ifosfamide/paclitaxel/cisplatin | 0.0% |
| Not applicable | 15.4% |
| Other (please specify) | 0.0% |
| 1. **For cisplatin-ineligible muscle invasive patients (cT2-T4aN0) do you recommend neoadjuvant chemotherapy?** | |
| **Answer Options** | **Response Percent** |
| Yes | 7.7% |
| No | 76.9% |
| Not applicable | 15.4% |
| 1. **Which of the following regimens do you commonly use or recommend for cisplatin-eligible patients as first-line therapy patients with metastatic bladder cancer? (select all that apply):** | |
| **Answer Options** | **Response Percent** |
| Gemcitabine/cisplatin | 69.2% |
| Dose dense gemcitabine/cisplatin | 23.1% |
| M-VAC | 15.4% |
| Dose dense M-VAC | 38.5% |
| Ifosfamide/paclitaxel/cisplatin | 0.0% |
| Not applicable | 15.4% |
| Other (please specify) | 7.7% |
| 1. **Which of the following regimens have you used or recommend for cisplatin-ineligible patients as first-line therapy patients with metastatic bladder cancer? (select all that apply):** | |
| **Answer Options** | **Response Percent** |
| Gemcitabine/carboplatin | 69.2% |
| M-CAVI | 7.7% |
| Gemcitabine monotherapy | 7.7% |
| Paclitaxel/Carboplatin | 15.4% |
| Gemcitabine/paclitaxel/doxorubicin | 23.1% |
| Not Applicable | 15.4% |
| Other (please specify) | 0.0% |
| 1. **Do you feel that immunotherapy is appropriate to test in front-line cisplatin-eligible patients as monotherapy (i.e. Instead of chemotherapy)?** | |
| **Answer Options** | **Response Percent** |
| Yes | 23.1% |
| No | 53.8% |
| No opinion | 23.1% |
| 1. **Do you feel that immunotherapy is appropriate to test in cisplatin-ineligible patients as monotherapy (i.e. instead of chemotherapy)?** | |
| **Answer Options** | **Response Percent** |
| Yes | 76.9% |
| No | 7.7% |
| No opinion | 15.4% |
| 1. **What is your “standard” second-line chemotherapy agent?:** | |
| **Answer Options** | **Response Percent** |
| Paclitaxel | 23.1% |
| Docetaxel | 23.1% |
| Pemetrexed | 15.4% |
| Ifosfamide | 0.0% |
| Vinorelbine | 0.0% |
| 5FU | 0.0% |
| Doxorubicin | 7.7% |
| Not applicable | 23.1% |
| Other (please specify) | 7.7% |
| 1. **What do you feel is an appropriate comparator for randomized 2nd-line trials?:** | |
| **Answer Options** | **Response Percent** |
| Pemetrexed | 7.7% |
| Paclitaxel | 23.1% |
| Docetaxel | 7.7% |
| Vinflunine (Europe) | 0.0% |
| Dealer’s choice | 38.5% |
| Not applicable | 23.1% |
| Other (please specify) | 0.0% |

*Note: The pre-meeting survey questions were answered prior to the approval of atezolizumab and nivolumab for advanced bladder cancer.
