# Supplementary material for: Society for Immunotherapy of Cancer consensus statement on immunotherapy for the treatment of bladder carcinoma
Source: J Immunother Cancer. 2017 Aug 15;5:68. doi: 10.1186/s40425-017-0271-0 (PMC5557323; doi:10.1186/s40425-017-0271-0)
Supplement: Supplementary file 4 — Cancer Immunotherapy Guidelines-Bladder Bibliography. The full bibliography (Cancer Immunotherapy Guidelines-Bladder Cancer). The literature search was performed as outlined in the Methods section of this manuscript (PDF 215 kb) [file 40425_2017_271_MOESM4_ESM.pdf]

CANCER IMMUNOTHERAPY GUIDELINES (BLADDER)

A Bibliography of the Literature (in order of topic)

SOCIETY FOR IMMUNOTHERAPY OF CANCER

JULY 11, 2017

Prepared by

silent PARTNERS
Comprehensive Resources. Clear Results.

8727 Shoal Creek Blvd. • Austin, TX 78757-6815

Phone: 512-458-1191 • Fax: 512-458-1234

www.silentpartners.com

JO21124

Table of Contents

TOPIC: Transitional Cell Carcinoma or Bladder Cancer or Urothelial Cancer and Atezolizumab 7

TOPIC: Transitional Cell Carcinoma or Bladder Cancer or Urothelial Cancer and BCG 7

TOPIC: Transitional Cell Carcinoma or Bladder Cancer or Urothelial Cancer and Combination Therapy 23

TOPIC: Transitional Cell Carcinoma or Bladder Cancer or Urothelial Cancer and Interferon 24

TOPIC: Transitional Cell Carcinoma or Bladder Cancer or Urothelial Cancer and Ipilimumab 25

TOPIC: Transitional Cell Carcinoma or Bladder Cancer or Urothelial Cancer and Nivolumab 25

TOPIC: Transitional Cell Carcinoma or Bladder Cancer or Urothelial Cancer and PD1/PD-L1 25

TOPIC: Transitional Cell Carcinoma or Bladder Cancer or Urothelial Cancer and Pembrolizumab 26

TOPIC: Author Additions 26

Note: This bibliography is organized in topic order and then in alphabetical order by author. The searches were originally conducted on 10/17/16 and then again on 6/17/17 and 7/8/17 to incorporate any additional references.

PREVIOUS SEARCHES

Bladder Cancer Immunotherapy Literature Search Conducted October 17, 2016										
**Search Terms**	**Date Limits**	**Limits**	**Search Field Tags**	**Data Search Completed**	**EndNote record numbers**	**Total records found**	**Total # Duplicates**	**Resulting # of records in bibliography**	**Total Drops**	
[Transitional cell carcinoma OR Bladder cancer OR Urothelial cancer] and BCG vaccine	1995-2016	(("carcinoma, transitional cell"[MeSH Terms] OR "urinary bladder neoplasms"[MeSH Terms]) AND "bcg vaccine"[MeSH Terms] AND (("1995/01/01"[PDAT] : "2016/12/31"[PDAT]) AND "humans"[MeSH Terms]) AND (Clinical Trial[ptyp] OR Controlled Clinical Trial[ptyp] OR Randomized Controlled Trial[ptyp] OR Meta-Analysis[ptyp] OR Practice Guideline[ptyp]))	MeSH Terms, Publication Date, Publication Type	10/17/2016	1-229* *Four references were inadvertently deleted and then added back in. The new reference numbers are 321 (formerly record number 85), 322 (formerly record number 32), 323 (formerly record number 22), and 324 (formerly record number 98).	229	0	229	87	142
[Transitional cell carcinoma OR Bladder cancer OR Urothelial cancer] and interferon	1995-2016	(("carcinoma, transitional cell"[MeSH Terms] OR "urinary bladder neoplasms"[MeSH Terms]) AND "Interferons"[MeSH Terms] AND (("1995/01/01"[PDAT] : "2016/12/31"[PDAT]) AND "humans"[MeSH Terms]) AND (Clinical Trial[ptyp] OR Controlled Clinical Trial[ptyp] OR Randomized Controlled Trial[ptyp] OR Meta-Analysis[ptyp] OR Practice Guideline[ptyp]))	MeSH Terms, Publication Date, Publication Type	10/17/2016	230-279	50	22	28	22	6

Bladder Cancer Immunotherapy Literature Search Conducted October 17, 2016										
**Search Terms**	**Date Limits**	**Limits**	**Search Field Tags**	**Data Search Completed**	**EndNote record numbers**	**Total records found**	**Total # Duplicates**	**Resulting # of records in bibliography**	**Total Drops**	
[Transitional cell carcinoma OR Bladder cancer OR Urothelial cancer] and ipilimumab	1995-2016	("carcinoma, transitional cell"[MeSH Terms] OR "urinary bladder neoplasms"[MeSH Terms]) AND "ipilimumab"[Supplementary Concept] AND (("1995/01/01"[PDAT] : "2014/12/31"[PDAT]) AND "humans"[MeSH Terms]) AND (Clinical Trial[ptyp] OR Controlled Clinical Trial[ptyp] OR Randomized Controlled Trial[ptyp] OR Meta-Analysis[ptyp] OR Practice Guideline[ptyp])	MeSH Terms, Publication Date, Publication Type	10/17/2016	280-282	3	1	2	0	2
Author Additions	N/A	Selected publications added by authors	N/A	N/A	283-319	37	1	36	0	36
Error	N/A	One entry was inadvertently added to the database and later dropped	N/A	N/A	320	1	0	1	1	0
					**Totals**	320	24	296	110	186

2017 SUPPLEMENTAL SEARCHES

Bladder Cancer Immunotherapy Supplemental Literature Searches Conducted June 17, 2017 and July 8, 2017												
Search Terms	Date Limits	Query Translation	Date Search Completed	Total Refs Identified	EndNote record numbers	Total records found	total # dupes	Total # Irrelevant Records	Resulting # of records in bibliography	New record # assignment when imported to original database	Total # Dupes	Resulting # of records in bibliography
transitional cell carcinoma OR bladder cancer OR urothelial cancer AND BCG	1/1/2015-12/31/17	((("carcinoma, transitional cell"[MeSH Terms] OR "urinary bladder neoplasms"[MeSH Terms]) OR urothelial[All Fields]) AND "neoplasms"[MeSH Terms]) AND BCG[All Fields] AND ((Clinical Trial[ptyp] OR Controlled Clinical Trial[ptyp] OR Meta-Analysis[ptyp] OR Practice Guideline[ptyp] OR Randomized Controlled Trial[ptyp]) AND ("2015/01/01"[PDAT] : "2017/12/31"[PDAT]) AND "humans"[MeSH Terms])	6/17/2017	23	1-23	23	0	10	13	325-337	4	9
transitional cell carcinoma OR bladder cancer OR urothelial cancer AND interferon	1/1/2015-12/31/17	((("carcinoma, transitional cell"[MeSH Terms] OR "urinary bladder neoplasms"[MeSH Terms]) OR urothelial[All Fields]) AND "neoplasms"[MeSH Terms]) AND "interferons"[MeSH Terms] AND ((Clinical Trial[ptyp] OR Controlled Clinical Trial[ptyp] OR Meta-Analysis[ptyp] OR Practice Guideline[ptyp] OR Randomized Controlled Trial[ptyp]) AND ("2015/01/01"[PDAT] : "2017/12/31"[PDAT]) AND "humans"[MeSH Terms])	6/17/2017	1	24	1	1		0			0
transitional cell carcinoma OR bladder cancer OR urothelial cancer AND ipilimumab	1/1/2015-12/31/17	((("carcinoma, transitional cell"[MeSH Terms] OR "urinary bladder neoplasms"[MeSH Terms]) OR urothelial[All Fields]) AND "neoplasms"[MeSH Terms]) AND "ipilimumab"[Supplementary Concept] AND ((Clinical Trial[ptyp] OR Controlled Clinical Trial[ptyp] OR Meta-Analysis[ptyp] OR Practice Guideline[ptyp] OR Randomized Controlled Trial[ptyp]) AND ("2015/01/01"[PDAT] : "2017/12/31"[PDAT]) AND "humans"[MeSH Terms])	6/17/2017	0		0			0			0
transitional cell carcinoma OR bladder cancer OR urothelial cancer AND nivolumab	1/1/2010-12/31/17	((("carcinoma, transitional cell"[MeSH Terms] OR "urinary bladder neoplasms"[MeSH Terms]) OR urothelial[All Fields]) AND "neoplasms"[MeSH Terms]) AND "nivolumab"[Supplementary Concept] AND ((Clinical Trial[ptyp] OR Controlled Clinical Trial[ptyp] OR Meta-Analysis[ptyp] OR Practice Guideline[ptyp] OR Randomized Controlled Trial[ptyp]) AND ("2010/01/01"[PDAT] : "2017/12/31"[PDAT]) AND "humans"[MeSH Terms])	6/17/2017	2	25-26	2	0		2	338-339	0	2
transitional cell carcinoma OR bladder cancer OR urothelial cancer AND pembrolizumab	1/1/2010-12/31/17	((("carcinoma, transitional cell"[MeSH Terms] OR "urinary bladder neoplasms"[MeSH Terms]) OR urothelial[All Fields]) AND "neoplasms"[MeSH Terms]) AND "pembrolizumab"[Supplementary Concept] AND ((Clinical Trial[ptyp] OR Controlled Clinical Trial[ptyp] OR Meta-Analysis[ptyp] OR Practice Guideline[ptyp] OR Randomized Controlled Trial[ptyp]) AND ("2010/01/01"[PDAT] : "2017/12/31"[PDAT]) AND "humans"[MeSH Terms])	6/17/2017	3	27-29	3	0	1	2	340-341	0	2
transitional cell carcinoma OR bladder cancer OR urothelial cancer AND atezolizumab	1/1/2010-12/31/17	((("carcinoma, transitional cell"[MeSH Terms] OR "urinary bladder neoplasms"[MeSH Terms]) OR urothelial[All Fields]) AND "neoplasms"[MeSH Terms]) AND "atezolizumab"[Supplementary Concept] AND ((Clinical Trial[ptyp] OR Controlled Clinical Trial[ptyp] OR Meta-Analysis[ptyp] OR Practice Guideline[ptyp] OR Randomized Controlled Trial[ptyp]) AND ("2010/01/01"[PDAT] : "2017/12/31"[PDAT]) AND "humans"[MeSH Terms])	6/17/2017	4	30-33	4	0	1	3	342-344	0	3

Bladder Cancer Immunotherapy Supplemental Literature Searches Conducted June 17, 2017 and July 8, 2017												
**Search Terms**	**Date Limits**	**Query Translation**	**Date Search Completed**	**Total Refs Identified**	**EndNote record numbers**	**Total records found**	**total # dupes**	**Total # Irrelevant Records**	**Resulting # of records in bibliography**	**New record # assignment when imported to original database**	**Total # Dupes**	**Resulting # of records in bibliography**
transitional cell carcinoma OR bladder cancer OR urothelial cancer AND durvalumab	1/1/2010-12/31/17	((("carcinoma, transitional cell"[MeSH Terms] OR "urinary bladder neoplasms"[MeSH Terms]) OR urothelial[All Fields]) AND "neoplasms"[MeSH Terms]) AND "durvalumab"[Supplementary Concept] AND ((Clinical Trial[ptyp] OR Controlled Clinical Trial[ptyp] OR Meta-Analysis[ptyp] OR Practice Guideline[ptyp] OR Randomized Controlled Trial[ptyp]) AND ("2010/01/01"[PDAT] : "2017/12/31"[PDAT]) AND "humans"[MeSH Terms])	6/17/2017	1	34	1	0	1	0			0
additional references added by Rosenbj1			7/8/2017	1	259	1			1	359	0	1
transitional cell carcinoma OR bladder cancer OR urothelial cancer AND avelumab	1/1/2010-12/31/17	((("carcinoma, transitional cell"[MeSH Terms] OR "urinary bladder neoplasms"[MeSH Terms]) OR urothelial[All Fields]) AND "neoplasms"[MeSH Terms]) AND "avelumab"[Supplementary Concept] AND ((Clinical Trial[ptyp] OR Controlled Clinical Trial[ptyp] OR Meta-Analysis[ptyp] OR Practice Guideline[ptyp] OR Randomized Controlled Trial[ptyp]) AND ("2010/01/01"[PDAT] : "2017/12/31"[PDAT]) AND "humans"[MeSH Terms])	6/17/2017	0		0			0		0	0
transitional cell carcinoma OR bladder cancer OR urothelial cancer AND PD1/PD-L1	1/1/2010-12/31/17	(((("carcinoma, transitional cell"[MeSH Terms] OR "urinary bladder neoplasms"[MeSH Terms]) OR urothelial[All Fields]) AND "neoplasms"[MeSH Terms]) AND PD1[All Fields]) OR ("antigens, cd274"[MeSH Terms] OR ("antigens"[All Fields] AND "cd274"[All Fields]) OR "cd274 antigens"[All Fields] OR ("pd"[All Fields] AND "l1"[All Fields]) OR "pd l1"[All Fields]) AND ((Clinical Trial[ptyp] OR Controlled Clinical Trial[ptyp] OR Meta-Analysis[ptyp] OR Practice Guideline[ptyp] OR Randomized Controlled Trial[ptyp]) AND ("2010/01/01"[PDAT] : "2017/12/31"[PDAT]) AND "humans"[MeSH Terms])	6/17/2017	91	35-125	91	8	82	1	345	0	1
transitional cell carcinoma OR bladder cancer OR urothelial cancer AND combination therapy	1/1/2010-12/31/17	((("carcinoma, transitional cell"[MeSH Terms] OR "urinary bladder neoplasms"[MeSH Terms]) OR urothelial[All Fields]) AND "neoplasms"[MeSH Terms]) AND "combined modality therapy"[MeSH Terms] AND ((Clinical Trial[ptyp] OR Controlled Clinical Trial[ptyp] OR Meta-Analysis[ptyp] OR Practice Guideline[ptyp] OR Randomized Controlled Trial[ptyp]) AND ("2010/01/01"[PDAT] : "2017/12/31"[PDAT]) AND "humans"[MeSH Terms])	6/17/2017	128	126-253	128	4	111	13	346-358	4	9
transitional cell carcinoma OR bladder cancer OR urothelial cancer AND peptide derived vaccine	1/1/2010-12/31/17	((("carcinoma, transitional cell"[MeSH Terms] OR "urinary bladder neoplasms"[MeSH Terms]) OR urothelial[All Fields]) AND "neoplasms"[MeSH Terms]) AND ("vaccines, subunit"[MeSH Terms] OR ("vaccines"[All Fields] AND "subunit"[All Fields]) OR "subunit vaccines"[All Fields] OR ("peptide"[All Fields] AND "vaccines"[All Fields]) OR "peptide vaccines"[All Fields]) AND ((Clinical Trial[ptyp] OR Controlled Clinical Trial[ptyp] OR Meta-Analysis[ptyp] OR Practice Guideline[ptyp] OR Randomized Controlled Trial[ptyp]) AND ("2010/01/01"[PDAT] : "2017/12/31"[PDAT]) AND "humans"[MeSH Terms])	6/17/2017	5	254-258	5	1	4	0			0
					TOTALS	259	14		35			27

TOPIC: Transitional Cell Carcinoma or Bladder Cancer or Urothelial Cancer and Atezolizumab

1 [342]. Balar, A. V., M. D. Galsky, et al. (2017). "Atezolizumab as first-line treatment in cisplatin-ineligible patients with locally advanced and metastatic urothelial carcinoma: a single-arm, multicentre, phase 2 trial." Lancet **389**(10064): 67-76.

2 [344]. Powles, T., J. P. Eder, et al. (2014). "MPDL3280A (anti-PD-L1) treatment leads to clinical activity in metastatic bladder cancer." Nature **515**(7528): 558-562.

3 [343]. Rosenberg, J. E., J. Hoffman-Censits, et al. (2016). "Atezolizumab in patients with locally advanced and metastatic urothelial carcinoma who have progressed following treatment with platinum-based chemotherapy: a single-arm, multicentre, phase 2 trial." Lancet **387**(10031): 1909-1920.

TOPIC: Transitional Cell Carcinoma or Bladder Cancer or Urothelial Cancer and BCG

1 [225]. Akaza, H., S. Hinotsu, et al. (1995). "Bacillus Calmette-Guerin treatment of existing papillary bladder cancer and carcinoma in situ of the bladder. Four-year results. The Bladder Cancer BCG Study Group." Cancer **75**(2): 552-559.

2 [123]. Akaza, H., K. Koiso, et al. (2003). "A clinical study of PMCJ-9 (Bacillus Calmette-Guerin Connaught strain) treatment of superficial bladder cancer and carcinoma in situ of the bladder." Jpn J Clin Oncol **33**(8): 382-390.

3 [28]. Babjuk, M., M. Burger, et al. (2013). "EAU guidelines on non-muscle-invasive urothelial carcinoma of the bladder: update 2013." Eur Urol **64**(4): 639-653.

4 [169]. Bassi, P., R. Spinadin, et al. (2000). "Modified induction course: a solution to side-effects?" Eur Urol **37 Suppl 1**: 31-32.

5 [155]. Bazarbashi, S., M. A. Raja, et al. (2000). "Prospective phase II trial of alternating intravesical Bacillus Calmette-Guerin (BCG) and interferon alpha IIB in the treatment and prevention of superficial transitional cell carcinoma of the urinary bladder: preliminary results." J Surg Oncol **74**(3): 181-184.

6 [54]. Bazarbashi, S., H. Soudy, et al. (2011). "Co-administration of intravesical

bacillus Calmette-Guerin and interferon alpha-2B as first line in treating superficial transitional cell carcinoma of the urinary bladder." BJU Int **108**(7): 1115-1118.

7 [80]. Beltrami, P., L. Ruggera, et al. (2008). "Are prostate biopsies mandatory in patients with prostate-specific antigen increase during intravesical immuno- or chemotherapy for superficial bladder cancer?" Prostate **68**(11): 1241-1247.

8 [207]. Berry, D. L., B. A. Blumenstein, et al. (1996). "Local toxicity patterns associated with intravesical bacillus Calmette-Guerin: a Southwest Oncology Group Study." Int J Urol **3**(2): 98-100; discussion 101.

9 [120]. Bohle, A. and P. R. Bock (2004). "Intravesical bacille Calmette-Guerin versus mitomycin C in superficial bladder cancer: formal meta-analysis of comparative studies on tumor progression." Urology **63**(4): 682-686; discussion 686-687.

10 [134]. Bohle, A., D. Jocham, et al. (2003). "Intravesical bacillus Calmette-Guerin versus mitomycin C for superficial bladder cancer: a formal meta-analysis of comparative studies on recurrence and toxicity." J Urol **169**(1): 90-95.

11 [160]. Bono, A. V., J. A. Lovisolo, et al. (2000). "Transurethral resection and sequential chemo-immunoprophylaxis in primary T1G3 bladder cancer." Eur Urol **37**(4): 478-483.

12 [159]. Brake, M., H. Loertzer, et al. (2000). "Long-term results of intravesical bacillus Calmette-Guerin therapy for stage T1 superficial bladder cancer." Urology **55**(5): 673-678.

13 [26]. Brausi, M., J. Oddens, et al. (2014). "Side effects of Bacillus Calmette-Guerin (BCG) in the treatment of intermediate- and high-risk Ta, T1 papillary carcinoma of the bladder: results of the EORTC genito-urinary cancers group randomised phase 3 study comparing one-third dose with full dose and 1 year with 3 years of maintenance BCG." Eur Urol **65**(1): 69-76.

14 [190]. Bui, T. T. and P. F. Schellhammer (1997). "Additional bacillus Calmette-Guerin therapy for recurrent transitional cell carcinoma after an initial complete response." Urology **49**(5): 687-690; discussion 690-681.

15 [79]. Cai, T., G. Nesi, et al. (2008). "Can early single dose instillation of epirubicin improve bacillus Calmette-Guerin efficacy in patients with nonmuscle invasive high risk bladder cancer? Results from a prospective, randomized, double-blind controlled study." J Urol **180**(1): 110-115.

16 [5]. Cambier, S., R. J. Sylvester, et al. (2016). "EORTC Nomograms and Risk Groups for Predicting Recurrence, Progression, and Disease-specific and Overall Survival in Non-Muscle-invasive Stage Ta-T1 Urothelial Bladder Cancer Patients Treated with 1-3 Years of Maintenance Bacillus Calmette-Guerin." Eur Urol **69**(1): 60-69.

17 [112]. Cheng, C. W., S. F. Chan, et al. (2005). "Twelve-year follow up of a randomized prospective trial comparing bacillus Calmette-Guerin and epirubicin as adjuvant therapy in superficial bladder cancer." Int J Urol **12**(5): 449-455.

18 [135]. Chepurov, A. K., E. Murshudli, et al. (2002). "[Features of BCG-therapy in treating patients with superficial bladder cancer]." Ter Arkh **74**(10): 70-72.

19 [96]. Colombel, M., F. Saint, et al. (2006). "The effect of ofloxacin on bacillus calmette-guerin induced toxicity in patients with superficial bladder cancer: results of a randomized, prospective, double-blind, placebo controlled, multicenter study." J Urol **176**(3): 935-939.

20 [330]. Cui, J., W. Wang, et al. (2016). "Combination of Intravesical Chemotherapy and Bacillus Calmette-Guerin Versus Bacillus Calmette-Guerin Monotherapy in Intermediate- and High-risk Nonmuscle Invasive Bladder Cancer: A Systematic Review and Meta-analysis." Medicine (Baltimore) **95**(3): e2572.

21 [172]. Dalbagni, G., T. Rechtschaffen, et al. (1999). "Is transurethral biopsy of the bladder necessary after 3 months to evaluate response to bacillus Calmette-Guerin therapy?" J Urol **162**(3 Pt 1): 708-709.

22 [74]. Damiano, R., M. De Sio, et al. (2009). "Short-term administration of prulifloxacin in patients with nonmuscle-invasive bladder cancer: an effective option for the prevention of bacillus Calmette-Guerin-induced toxicity?" BJU Int **104**(5): 633-639.

23 [154]. de Boer, E. C., T. M. De Reijke, et al. (2000). "Immunostimulation in the urinary bladder by local application of Nocardia rubra cell-wall skeletons (Rubratin) and bacillus Calmette-Guerin as therapy for superficial bladder cancer: a comparative study." Clin Infect Dis **31 Suppl 3**: S109-114.

24 [210]. de Reijke, T. M., E. C. de Boer, et al. (1996). "Urinary cytokines during intravesical bacillus Calmette-Guerin therapy for superficial bladder cancer: processing, stability and prognostic value." J Urol **155**(2): 477-482.

25 [179]. de Reijke, T. M., E. C. De Boer, et al. (1999). "Urinary interleukin-2 monitoring during prolonged bacillus Calmette-Guerin treatment: can it predict the optimal number of instillations?" J Urol **161**(1): 67-71.

26 [197]. de Reijke, T. M., E. C. de Boer, et al. (1997). "Immunostimulation in the urinary bladder by local application of Nocardia rubra cell wall skeleton preparation (Rubratin) for superficial bladder cancer immunotherapy--a phase I/II study." Urol Res **25**(2): 117-120.

27 [114]. de Reijke, T. M., K. H. Kurth, et al. (2005). "Bacillus Calmette-Guerin versus epirubicin for primary, secondary or concurrent carcinoma in situ of the bladder: results of a European Organization for the Research and Treatment of Cancer--Genito-Urinary Group Phase III Trial (30906)." J Urol **173**(2): 405-409.

28 [78]. Decobert, M., H. LaRue, et al. (2008). "Maintenance bacillus Calmette-Guerin in high-risk nonmuscle-invasive bladder cancer: how much is enough?" Cancer **113**(4): 710-716.

29 [66]. Di Lorenzo, G., S. Perdona, et al. (2010). "Gemcitabine versus bacille Calmette-Guerin after initial bacille Calmette-Guerin failure in non-muscle-invasive bladder cancer: a multicenter prospective randomized trial." Cancer **116**(8): 1893-1900.

30 [105]. Di Stasi, S. M., A. Giannantoni, et al. (2006). "Sequential BCG and electromotive mitomycin versus BCG alone for high-risk superficial bladder cancer: a randomised controlled trial." Lancet Oncol **7**(1): 43-51.

31 [108]. Ding, G. Q., Z. J. Shen, et al. (2005). "[Clinical investigation on the effect of intravesical instillation of antifibrinolytic agents with bacillus Calmette-Guerin on preventing bladder cancer recurrence]." Zhonghua Wai Ke Za Zhi **43**(22): 1457-1460.

32 [33]. Dinney, C. P., M. B. Fisher, et al. (2013). "Phase I trial of intravesical recombinant adenovirus mediated interferon-alpha2b formulated in Syn3 for Bacillus Calmette-Guerin failures in nonmuscle invasive bladder cancer." J Urol **190**(3): 850-856.

33 [69]. Duchek, M., R. Johansson, et al. (2010). "Bacillus Calmette-Guerin is superior to a combination of epirubicin and interferon-alpha2b in the intravesical treatment of patients with stage T1 urinary bladder cancer. A prospective, randomized, Nordic study." Eur Urol **57**(1): 25-31.

34 [24]. Farah, N. B., R. Ghanem, et al. (2014). "Treatment efficacy and tolerability of intravesical bacillus Calmette-Guerin (BCG)-RIVM strain: induction and maintenance protocol in high grade and recurrent low grade non-muscle invasive bladder cancer (NMIBC)." BMC Urol **14**: 11.

35 [92]. Friedrich, M. G., U. Pichlmeier, et al. (2007). "Long-term intravesical adjuvant chemotherapy further reduces recurrence rate compared with short-term intravesical chemotherapy and short-term therapy with Bacillus Calmette-Guerin (BCG) in patients with non-muscle-invasive bladder carcinoma." Eur Urol **52**(4): 1123-1129.

36 [83]. Gallagher, B. L., F. N. Joudi, et al. (2008). "Impact of previous bacille Calmette-Guerin failure pattern on subsequent response to bacille Calmette-Guerin plus interferon intravesical therapy." Urology **71**(2): 297-301.

37 [32]. Galliot, I., S. Le Gall, et al. (2013). "[Bacillus Calmette-Guerin maintenance treatment in non-invasive bladder tumors: 1 year follow-up results of multicenter URO-BCG-4 trial]." Prog Urol **23**(5): 336-346.

38 [178]. Gan, Y. H., R. Mahendran, et al. (1999). "Evaluation of lymphocytic responses after treatment with Bacillus Calmette-Guerin and interferon-alpha 2b for superficial bladder cancer." Clin Immunol **90**(2): 230-237.

39 [94]. Gardmark, T., S. Jahnson, et al. (2007). "Analysis of progression and survival after 10 years of a randomized prospective study comparing mitomycin-C and bacillus Calmette-Guerin in patients with high-risk bladder cancer." BJU Int **99**(4): 817-820.

40 [31]. Gontero, P., M. Oderda, et al. (2013). "The impact of intravesical gemcitabine and 1/3 dose Bacillus Calmette-Guerin instillation therapy on the quality of life in patients with nonmuscle invasive bladder cancer: results of a prospective, randomized, phase II trial." J Urol **190**(3): 857-862.

41 [195]. Gruenwald, I. E., A. Stein, et al. (1997). "A 12 versus 6-week course of bacillus Calmette-Guerin prophylaxis for the treatment of high risk superficial bladder cancer." J Urol **157**(2): 487-491.

42 [43]. Gulpinar, O., A. H. Halilioglu, et al. (2012). "The value of perioperative mitomycin C instillation in improving subsequent bacillus calmette-guerin instillation efficacy in intermediate and high-risk patients with non-muscle invasive bladder cancer: a prospective randomized study." Int Braz J Urol **38**(4): 474-479.

43 [321]. Guy, L., L. Savareux, et al. (2006). "Should bladder biopsies be performed routinely after bacillus Calmette-Guerin treatment for high-risk superficial transitional cell cancer of the bladder?" Eur Urol **50**(3): 516-520; discussion 520.

44 [99]. Han, R. F. and J. G. Pan (2006). "Can intravesical bacillus Calmette-Guerin reduce recurrence in patients with superficial bladder cancer?

A meta-analysis of randomized trials." Urology **67**(6): 1216-1223.

45 [119]. Hayashida, Y., K. Nomata, et al. (2004). "Long-term effects of bacille Calmette-Guerin perfusion therapy for treatment of transitional cell carcinoma in situ of upper urinary tract." Urology **63**(6): 1084-1088.

46 [7]. Hayne, D., M. Stockler, et al. (2015). "BCG+MMC trial: adding mitomycin C to BCG as adjuvant intravesical therapy for high-risk, non-muscle-invasive bladder cancer: a randomised phase III trial (ANZUP 1301)." BMC Cancer **15**: 432.

47 [25]. Hemdan, T., R. Johansson, et al. (2014). "5-Year outcome of a randomized prospective study comparing bacillus Calmette-Guerin with epirubicin and interferon-alpha2b in patients with T1 bladder cancer." J Urol **191**(5): 1244-1249.

48 [185]. Herr, H. W. (1997). "Tumour progression and survival in patients with T1G3 bladder tumours: 15-year outcome." Br J Urol **80**(5): 762-765.

49 [220]. Herr, H. W., D. M. Schwalb, et al. (1995). "Intravesical bacillus Calmette-Guerin therapy prevents tumor progression and death from superficial bladder cancer: ten-year follow-up of a prospective randomized trial." J Clin Oncol **13**(6): 1404-1408.

50 [102]. Hinotsu, S., H. Akaza, et al. (2006). "Sustained prophylactic effect of intravesical bacille Calmette-Guerin for superficial bladder cancer: a smoothed hazard analysis in a randomized prospective study." Urology **67**(3): 545-549.

51 [56]. Hinotsu, S., H. Akaza, et al. (2011). "Maintenance therapy with bacillus Calmette-Guerin Connaught strain clearly prolongs recurrence-free survival following transurethral resection of bladder tumour for non-muscle-invasive bladder cancer." BJU Int **108**(2): 187-195.

52 [36]. Houghton, B. B., V. Chalasani, et al. (2013). "Intravesical chemotherapy plus bacille Calmette-Guerin in non-muscle invasive bladder cancer: a systematic review with meta-analysis." BJU Int **111**(6): 977-983.

53 [128]. Huncharek, M. and B. Kupelnick (2003). "Impact of intravesical chemotherapy versus BCG immunotherapy on recurrence of superficial transitional cell carcinoma of the bladder: metaanalytic reevaluation." Am J Clin Oncol **26**(4): 402-407.

54 [116]. Huncharek, M. and B. Kupelnick (2004). "The influence of intravesical therapy on progression of superficial transitional cell carcinoma of the bladder: a metaanalytic comparison of chemotherapy versus bacilli Calmette-Guerin

immunotherapy." Am J Clin Oncol **27**(5): 522-528.

55 [173]. Hurle, R., A. Losa, et al. (1999). "Intravesical bacille Calmette-Guerin in Stage T1 grade 3 bladder cancer therapy: a 7-year follow-up." Urology **54**(2): 258-263.

56 [199]. Hurle, R., A. Losa, et al. (1996). "Low dose Pasteur bacillus Calmette-Guerin regimen in stage T1, grade 3 bladder cancer therapy." J Urol **156**(5): 1602-1605.

57 [82]. Isbarn, H., L. Budaus, et al. (2008). "[Comparison of the effectiveness between long-term instillation of mitomycin C and short-term prophylaxis with MMC or bacille Calmette-Guerin. Study of patients with non-muscle-invasive urothelial cancer of the urinary bladder]." Urologe A **47**(5): 608-615.

58 [117]. Iturralde Codina, A., W. Beyrie Tamayo, et al. (2004). "[Bacillus Calmette Guerin as adjuvant treatment for superficial bladder tumors using two different therapeutic schemes]." Arch Esp Urol **57**(6): 606-618.

59 [147]. Jakse, G., R. Hall, et al. (2001). "Intravesical BCG in patients with carcinoma in situ of the urinary bladder: long-term results of EORTC GU Group phase II protocol 30861." Eur Urol **40**(2): 144-150.

60 [46]. Jarvinen, R., E. Kaasinen, et al. (2012). "Long-term results of maintenance treatment of mitomycin C or alternating mitomycin C and bacillus Calmette-Guerin instillation therapy of patients with carcinoma in situ of the bladder: a subgroup analysis of the prospective FinnBladder 2 study with a 17-year follow-up." Scand J Urol Nephrol **46**(6): 411-417.

61 [9].Jarvinen, R., T. Marttila, et al. (2015). "Long-term outcome of patients with frequently recurrent non-muscle-invasive bladder carcinoma treated with one perioperative plus four weekly instillations of mitomycin C followed by monthly bacillus Calmette-Guerin (BCG) or alternating BCG and interferon-alpha2b instillations: prospective randomised FinnBladder-4 study." Eur Urol **68**(4): 611-617.

62 [186]. Jimenez-Cruz, J. F., C. D. Vera-Donoso, et al. (1997). "Intravesical immunoprophylaxis in recurrent superficial bladder cancer (Stage T1): multicenter trial comparing bacille Calmette-Guerin and interferon-alpha." Urology **50**(4): 529-535.

63 [76]. Jocham, D., J. von Wietersheim, et al. (2009). "[BCG versus photodynamic therapy (PDT) for nonmuscle invasive bladder cancer-a multicentre clinical phase III study]." Aktuelle Urol **40**(2): 91-99.

64 [38]. Johnson, M. H., K. G. Nepple, et al. (2013). "Randomized controlled trial of oxybutynin extended release versus placebo for urinary symptoms during intravesical Bacillus Calmette-Guerin treatment." J Urol **189**(4): 1268-1274.

65 [97]. Joudi, F. N., B. J. Smith, et al. (2006). "Final results from a national multicenter phase II trial of combination bacillus Calmette-Guerin plus interferon alpha-2B for reducing recurrence of superficial bladder cancer." Urol Oncol **24**(4): 344-348.

66 [215]. Jurincic-Winkler, C., K. A. Metz, et al. (1995). "Effect of keyhole limpet hemocyanin (KLH) and bacillus Calmette-Guerin (BCG) instillation on carcinoma in situ of the urinary bladder." Anticancer Res **15**(6b): 2771-2776.

67 [138]. Kaasinen, E., E. Rintala, et al. (2002). "Factors explaining recurrence in patients undergoing chemoimmunotherapy regimens for frequently recurring superficial bladder carcinoma." Eur Urol **42**(2): 167-174.

68 [156]. Kaasinen, E., E. Rintala, et al. (2000). "Weekly mitomycin C followed by monthly bacillus Calmette-Guerin or alternating monthly interferon-alpha2B and bacillus Calmette-Guerin for prophylaxis of recurrent papillary superficial bladder carcinoma." J Urol **164**(1): 47-52.

69 [131]. Kaasinen, E., H. Wijkstrom, et al. (2003). "Alternating mitomycin C and BCG instillations versus BCG alone in treatment of carcinoma in situ of the urinary bladder: a nordic study." Eur Urol **43**(6): 637-645.

70 [326]. Kaasinen, E., H. Wijkstrom, et al. (2016). "Seventeen-year follow-up of the prospective randomized Nordic CIS study: BCG monotherapy versus alternating therapy with mitomycin C and BCG in patients with carcinoma in situ of the urinary bladder." Scand J Urol **50**(5): 360-368.

71 [322]. Kakiashvili, D. M., B. W. van Rhijn, et al. (2011). "Long-term follow-up of T1 high-grade bladder cancer after intravesical bacille Calmette-Guerin treatment." BJU Int **107**(4): 540-546.

72 [6].Kamat, A. M., J. Briggman, et al. (2016). "Cytokine Panel for Response to Intravesical Therapy (CyPRIT): Nomogram of Changes in Urinary Cytokine Levels Predicts Patient Response to Bacillus Calmette-Guerin." Eur Urol **69**(2): 197-200.

73 [48]. Kamat, A. M., R. J. Dickstein, et al. (2012). "Use of fluorescence in situ hybridization to predict response to bacillus Calmette-Guerin therapy for bladder cancer: results of a prospective trial." J Urol **187**(3): 862-867.

74 [323]. Kanagawa Urological Research, G. (2012). "A 2-week maintenance

regimen of intravesical instillation of bacillus Calmette-Guerin is safe, adherent and effective in patients with non-muscle-invasive bladder cancer: a prospective, multicenter phase II clinical trial." Jpn J Clin Oncol **42**(9): 813-819.

75 [115]. Kipp, B. R., R. J. Karnes, et al. (2005). "Monitoring intravesical therapy for superficial bladder cancer using fluorescence in situ hybridization." J Urol **173**(2): 401-404.

76 [324]. Koga, H., M. Kuroda, et al. (2005). "Adverse drug reactions of intravesical bacillus Calmette-Guerin instillation and risk factors of the development of adverse drug reactions in superficial cancer and carcinoma in situ of the bladder." Int J Urol **12**(2): 145-151.

77 [64]. Koga, H., S. Ozono, et al. (2010). "Maintenance intravesical bacillus Calmette-Guerin instillation for Ta, T1 cancer and carcinoma in situ of the bladder: randomized controlled trial by the BCG Tokyo Strain Study Group." Int J Urol **17**(9): 759-766.

78 [142]. Kolodziej, A., J. Dembowski, et al. (2002). "Treatment of high-risk superficial bladder cancer with maintenance bacille Calmette-Guerin therapy: preliminary results." BJU Int **89**(6): 620-622.

79 [60]. Kowalski, M., J. Entwistle, et al. (2010). "A Phase I study of an intravesically administered immunotoxin targeting EpCAM for the treatment of nonmuscle-invasive bladder cancer in BCGrefractory and BCG-intolerant patients." Drug Des Devel Ther **4**: 313-320.

80 [40]. Kowalski, M., J. Guindon, et al. (2012). "A phase II study of oportuzumab monatox: an immunotoxin therapy for patients with noninvasive urothelial carcinoma in situ previously treated with bacillus Calmette-Guerin." J Urol **188**(5): 1712-1718.

81 [204]. Krege, S., G. Giani, et al. (1996). "A randomized multicenter trial of adjuvant therapy in superficial bladder cancer: transurethral resection only versus transurethral resection plus mitomycin C versus transurethral resection plus bacillus Calmette-Guerin. Participating Clinics." J Urol **156**(3): 962-966.

82 [162]. Lamm, D. L., B. A. Blumenstein, et al. (2000). "Maintenance bacillus Calmette-Guerin immunotherapy for recurrent TA, T1 and carcinoma in situ transitional cell carcinoma of the bladder: a randomized Southwest Oncology Group Study." J Urol **163**(4): 1124-1129.

83 [166]. Lebret, T., D. Bohin, et al. (2000). "Recurrence, progression and success in stage Ta grade 3 bladder tumors treated with low dose bacillus

Calmette-Guerin instillations." J Urol **163**(1): 63-67.

84 [327]. Lenormand, C., J. Couteau, et al. (2016). "Predictive Value of NRAMP1 and HGPX1 Gene Polymorphism for Maintenance BCG Response in Non-muscle-invasive Bladder Cancer." Anticancer Res **36**(4): 1737-1743.

85 [91]. Lerner, S. P., C. M. Tangen, et al. (2007). "Patterns of recurrence and outcomes following induction bacillus Calmette-Guerin for high risk Ta, T1 bladder cancer." J Urol **177**(5): 1727-1731.

86 [81]. Lerner, S. P., C. M. Tangen, et al. (2009). "Failure to achieve a complete response to induction BCG therapy is associated with increased risk of disease worsening and death in patients with high risk non-muscle invasive bladder cancer." Urol Oncol **27**(2): 155-159.

87 [132]. Librenjak, D., M. Situm, et al. (2003). "Immunoprophylactic intravesical application of bacillus Calmette-Guerin after transurethral resection of superficial bladder cancer." Croat Med J **44**(2): 187-192.

88 [165]. Losa, A., R. Hurle, et al. (2000). "Low dose bacillus Calmette-Guerin for carcinoma in situ of the bladder: long-term results." J Urol **163**(1): 68-71; discussion 71-62.

89 [209]. Luftenegger, W., D. K. Ackermann, et al. (1996). "Intravesical versus intravesical plus intradermal bacillus Calmette-Guerin: a prospective randomized study in patients with recurrent superficial bladder tumors." J Urol **155**(2): 483-487.

90 [205]. Lundholm, C., B. J. Norlen, et al. (1996). "A randomized prospective study comparing long-term intravesical instillations of mitomycin C and bacillus Calmette-Guerin in patients with superficial bladder carcinoma." J Urol **156**(2 Pt 1): 372-376.

91 [224]. Mack, D. and J. Frick (1995). "Low-dose bacille Calmette-Guerin (BCG) therapy in superficial high-risk bladder cancer: a phase II study with the BCG strain Connaught Canada." Br J Urol **75**(2): 185-187.

92 [152]. Mack, D., W. Holtl, et al. (2001). "The ablative effect of quarter dose bacillus Calmette-Guerin on a papillary marker lesion of the bladder." J Urol **165**(2): 401-403.

93 [72]. Malmstrom, P. U., R. J. Sylvester, et al. (2009). "An individual patient data meta-analysis of the long-term outcome of randomised studies comparing intravesical mitomycin C versus bacillus Calmette-Guerin for non-muscle-invasive bladder cancer." Eur Urol **56**(2): 247-256.

94 [177]. Malmstrom, P. U., H. Wijkstrom, et al. (1999). "5-year followup of a randomized prospective study comparing mitomycin C and bacillus Calmette-Guerin in patients with superficial bladder carcinoma. Swedish-Norwegian Bladder Cancer Study Group." J Urol **161**(4): 1124-1127.

95 [140]. Martinez-Pineiro, J. A., N. Flores, et al. (2002). "Long-term follow-up of a randomized prospective trial comparing a standard 81 mg dose of intravesical bacille Calmette-Guerin with a reduced dose of 27 mg in superficial bladder cancer." BJU Int **89**(7): 671-680.

96 [111]. Martinez-Pineiro, J. A., L. Martinez-Pineiro, et al. (2005). "Has a 3-fold decreased dose of bacillus Calmette-Guerin the same efficacy against recurrences and progression of T1G3 and Tis bladder tumors than the standard dose? Results of a prospective randomized trial." J Urol **174**(4 Pt 1): 1242-1247.

97 [229]. Martinez-Pineiro, J. A., E. Solsona, et al. (1995). "Improving the safety of BCG immunotherapy by dose reduction. Cooperative Group CUETO." Eur Urol **27 Suppl 1**: 13-18.

98 [8]. Martinez-Pineiro, L., J. A. Portillo, et al. (2015). "Maintenance Therapy with 3-monthly Bacillus Calmette-Guerin for 3 Years is Not Superior to Standard Induction Therapy in High-risk Non-muscle-invasive Urothelial Bladder Carcinoma: Final Results of Randomised CUETO Study 98013." Eur Urol **68**(2): 256-262.

99 [17]. McKiernan, J. M., D. D. Holder, et al. (2014). "Phase II trial of intravesical nanoparticle albumin bound paclitaxel for the treatment of nonmuscle invasive urothelial carcinoma of the bladder after bacillus Calmette-Guerin treatment failure." J Urol **192**(6): 1633-1638.

100 [62]. Nepple, K. G., A. J. Lightfoot, et al. (2010). "Bacillus Calmette-Guerin with or without interferon alpha-2b and megadose versus recommended daily allowance vitamins during induction and maintenance intravesical treatment of nonmuscle invasive bladder cancer." J Urol **184**(5): 1915-1919.

101 [153]. Nonomura, N., Y. Ono, et al. (2000). "Bacillus Calmette-Guerin perfusion therapy for the treatment of transitional cell carcinoma in situ of the upper urinary tract." Eur Urol **38**(6): 701-704;discussion 705.

102 [37]. Oddens, J., M. Brausi, et al. (2013). "Final results of an EORTC-GU cancers group randomized study of maintenance bacillus Calmette-Guerin in intermediate- and high-risk Ta, T1 papillary carcinoma of the urinary bladder: one-third dose versus full dose and 1 year versus 3 years of maintenance." Eur Urol **63**(3): 462-472.

103 [329]. Oddens, J. R., R. J. Sylvester, et al. (2016). "Increasing age is not associated with toxicity leading to discontinuation of treatment in patients with urothelial non-muscle-invasive bladder cancer randomised to receive 3 years of maintenance bacille Calmette-Guerin: results from European Organisation for Research and Treatment of Cancer Genito-Urinary Group study 30911." BJU Int **118**(3): 423-428.

104 [18]. Oddens, J. R., R. J. Sylvester, et al. (2014). "The effect of age on the efficacy of maintenance bacillus Calmette-Guerin relative to maintenance epirubicin in patients with stage Ta T1 urothelial bladder cancer: results from EORTC genito-urinary group study 30911." Eur Urol **66**(4): 694-701.

105 [118]. O'Donnell, M. A., K. Lilli, et al. (2004). "Interim results from a national multicenter phase II trial of combination bacillus Calmette-Guerin plus interferon alfa-2b for superficial bladder cancer." J Urol **172**(3): 888-893.

106 [90]. Ojea, A., J. L. Nogueira, et al. (2007). "A multicentre, randomised prospective trial comparing three intravesical adjuvant therapies for intermediate-risk superficial bladder cancer: low-dose bacillus Calmette-Guerin (27 mg) versus very low-dose bacillus Calmette-Guerin (13.5 mg) versus mitomycin C." Eur Urol **52**(5): 1398-1406.

107 [203]. Okamura, T., K. Tozawa, et al. (1996). "Clinicopathological evaluation of repeated courses of intravesical bacillus Calmette-Guerin instillation for preventing recurrence of initially resistant superficial bladder cancer." J Urol **156**(3): 967-971.

108 [59]. Oosterlinck, W., Z. Kirkali, et al. (2011). "Sequential intravesical chemoimmunotherapy with mitomycin C and bacillus Calmette-Guerin and with bacillus Calmette-Guerin alone in patients with carcinoma in situ of the urinary bladder: results of an EORTC genito-urinary group randomized phase 2 trial (30993)." Eur Urol **59**(3): 438-446.

109 [335]. Orsola, A., L. Werner, et al. (2015). "Reexamining treatment of high-grade T1 bladder cancer according to depth of lamina propria invasion: a prospective trial of 200 patients." Br J Cancer **112**(3): 468-474.

110 [191]. Ovesen, H., T. Horn, et al. (1997). "Long-term efficacy of intravesical bacillus Calmette-Guerin for carcinoma in situ: relationship of progression to histological response and p53 nuclear accumulation." J Urol **157**(5): 1655-1659.

111 [149]. Palou, J., P. Laguna, et al. (2001). "Control group and maintenance treatment with bacillus Calmette-Guerin for carcinoma in situ and/or high grade

bladder tumors." J Urol **165**(5): 1488-1491.

112 [44]. Palou, J., D. Wood, et al. (2013). "ICUD-EAU International Consultation on Bladder Cancer 2012: Urothelial carcinoma of the prostate." Eur Urol **63**(1): 81-87.

113 [84]. Pan, C. W., Z. J. Shen, et al. (2008). "The effect of intravesical instillation of antifibrinolytic agents on bacillus Calmette-Guerin treatment of superficial bladder cancer: a pilot study." J Urol **179**(4): 1307-1311; discussion 1311-1302.

114 [175]. Pfister, C., J. M. Flaman, et al. (1999). "p53 mutations in bladder tumors inactivate the transactivation of the p21 and Bax genes, and have a predictive value for the clinical outcome after bacillus Calmette-Guerin therapy." J Urol **162**(1): 69-73.

115 [14]. Pfister, C., W. Kerkeni, et al. (2015). "Efficacy and tolerance of one-third full dose bacillus Calmette-Guerin maintenance therapy every 3 months or 6 months: two-year results of URO-BCG-4 multicenter study." Int J Urol **22**(1): 53-60.

116 [337]. Pfister, C., W. Kerkeni, et al. (2015). "Efficacy and tolerance of one-third full dose bacillus Calmette-Guerin maintenance therapy every 3 months or 6 months: two-year results of URO-BCG-4 multicenter study." Int J Urol **22**(1): 53-60.

117 [129]. Pieras, E., J. Palou, et al. (2003). "Management and prognosis of transitional cell carcinoma superficial recurrence in muscle-invasive bladder cancer after bladder preservation." Eur Urol **44**(2): 222-225; discussoion 225.

118 [328]. Rexer, H. (2016). "[A study of non-muscle-invasive bladder cancer therapy : Treatment of high-grade non-muscle-invasive urothelial carcinoma of the bladder with the standard number and dosage of intravesical BCG instillations versus a reduced number intravesical BCG instillations at the standard dosage: A European Association of Urology Research Foundation randomized phase 3 study - NIMBUS - AB 37/10 of the AUO]." Urologe A **55**(4): 528-531.

119 [206]. Rintala, E., K. Jauhiainen, et al. (1996). "Alternating mitomycin C and bacillus Calmette-Guerin instillation prophylaxis for recurrent papillary (stages Ta to T1) superficial bladder cancer. Finnbladder Group." J Urol **156**(1): 56-59; discussion 59-60.

120 [214]. Rintala, E., K. Jauhiainen, et al. (1995). "Alternating mitomycin C and bacillus Calmette-Guerin instillation therapy for carcinoma in situ of the bladder.

The Finnbladder Group." J Urol **154**(6): 2050-2053.

121 [50]. Rosevear, H. M., A. J. Lightfoot, et al. (2011). "Factors affecting response to bacillus Calmette-Guerin plus interferon for urothelial carcinoma in situ." J Urol **186**(3): 817-823.

122 [63]. Rosevear, H. M., A. J. Lightfoot, et al. (2010). "Safety and efficacy of intravesical bacillus Calmette-Guerin plus interferon alpha-2b therapy for nonmuscle invasive bladder cancer in patients with prosthetic devices." J Urol **184**(5): 1920-1924.

123 [325]. Roupret, M., Y. Neuzillet, et al. (2016). "[CCAFU french national guidelines 2016-2018 on bladder cancer]." Prog Urol **27 Suppl 1**: S67-S91.

124 [184]. Sarosdy, M. F., M. J. Manyak, et al. (1998). "Oral bropirimine immunotherapy of bladder carcinoma in situ after prior intravesical bacille Calmette-Guerin." Urology **51**(2): 226-231.

125 [109]. Sarosdy, M. F., C. M. Tangen, et al. (2005). "A phase II clinical trial of oral bropirimine in combination with intravesical bacillus Calmette-Guerin for carcinoma in situ of the bladder: a Southwest Oncology Group Study." Urol Oncol **23**(6): 386-389.

126 [227]. Schellhammer, P. F., L. E. Ladaga, et al. (1995). "Intravesical bacillus Calmette-Guerin for the treatment of superficial transitional cell carcinoma of the prostatic urethra in association with carcinoma of the bladder." J Urol **153**(1): 53-56.

127 [55]. Serretta, V., A. Ruggirello, et al. (2010). "[Prevention of topic toxicity of BCG with single-dose prulifloxacin. Preliminary results of a randomized pilot study]." Urologia **77**(4): 240-247.

128 [52]. Shang, P. F., J. Kwong, et al. (2011). "Intravesical Bacillus Calmette-Guerin versus epirubicin for Ta and T1 bladder cancer." Cochrane Database Syst Rev(5): Cd006885.

129 [3]. Shelley, M., J. B. Court, et al. (2015). "WITHDRAWN: Intravesical Bacillus Calmette-Guerin versus mitomycin C for Ta and T I bladder cancer." Cochrane Database Syst Rev(11): Cd003231.

130 [127]. Shelley, M. D., J. B. Court, et al. (2003). "Intravesical bacillus Calmette-Guerin versus mitomycin C for Ta and T1 bladder cancer." Cochrane Database Syst Rev(3): Cd003231.

131 [121]. Shelley, M. D., T. J. Wilt, et al. (2004). "Intravesical bacillus Calmette-Guerin is superior to mitomycin C in reducing tumour recurrence in high-risk superficial bladder cancer: a meta-analysis of randomized trials." BJU Int **93**(4): 485-490.

132 [30]. Skinner, E. C., B. Goldman, et al. (2013). "SWOG S0353: Phase II trial of intravesical gemcitabine in patients with nonmuscle invasive bladder cancer and recurrence after 2 prior courses of intravesical bacillus Calmette-Guerin." J Urol **190**(4): 1200-1204.

133 [13]. Solsona, E., R. Madero, et al. (2015). "Sequential combination of mitomycin C plus bacillus Calmette-Guerin (BCG) is more effective but more toxic than BCG alone in patients with non-muscle-invasive bladder cancer in intermediate- and high-risk patients: final outcome of CUETO 93009, a randomized prospective trial." Eur Urol **67**(3): 508-516.

134 [163]. Steinberg, G., R. Bahnson, et al. (2000). "Efficacy and safety of valrubicin for the treatment of Bacillus Calmette-Guerin refractory carcinoma in situ of the bladder. The Valrubicin Study Group." J Urol **163**(3): 761-767.

135 [336]. Svatek, R. S., X. R. Zhao, et al. (2015). "Sequential intravesical mitomycin plus Bacillus Calmette-Guerin for non-muscle-invasive urothelial bladder carcinoma: translational and phase I clinical trial." Clin Cancer Res **21**(2): 303-311.

136 [67]. Sylvester, R. J., M. A. Brausi, et al. (2010). "Long-term efficacy results of EORTC genito-urinary group randomized phase 3 study 30911 comparing intravesical instillations of epirubicin, bacillus Calmette-Guerin, and bacillus Calmette-Guerin plus isoniazid in patients with intermediate- and high-risk stage Ta T1 urothelial carcinoma of the bladder." Eur Urol **57**(5): 766-773.

137 [137]. Sylvester, R. J., M. A. van der, et al. (2002). "Intravesical bacillus Calmette-Guerin reduces the risk of progression in patients with superficial bladder cancer: a meta-analysis of the published results of randomized clinical trials." J Urol **168**(5): 1964-1970.

138 [125]. Sylvester, R. J., A. P. van der Meijden, et al. (2003). "The side effects of Bacillus Calmette-Guerin in the treatment of Ta T1 bladder cancer do not predict its efficacy: results from a European Organisation for Research and Treatment of Cancer Genito-Urinary Group Phase III Trial." Eur Urol **44**(4): 423-428.

139 [113]. Sylvester, R. J., A. P. van der Meijden, et al. (2005). "Bacillus calmette-guerin versus chemotherapy for the intravesical treatment of patients with carcinoma in situ of the bladder: a meta-analysis of the published results of

randomized clinical trials." J Urol **174**(1): 86-91; discussion 91-82.

140 [148]. van der Meijden, A. P., M. Brausi, et al. (2001). "Intravesical instillation of epirubicin, bacillus Calmette-Guerin and bacillus Calmette-Guerin plus isoniazid for intermediate and high risk Ta, T1 papillary carcinoma of the bladder: a European Organization for Research and Treatment of Cancer genito-urinary group randomized phase III trial." J Urol **166**(2): 476-481.

141 [124]. van der Meijden, A. P., R. J. Sylvester, et al. (2003). "Maintenance Bacillus Calmette-Guerin for Ta T1 bladder tumors is not associated with increased toxicity: results from a European Organisation for Research and Treatment of Cancer Genito-Urinary Group Phase III Trial." Eur Urol **44**(4): 429-434.

142 [222]. Vasavada, S. P., S. B. Streem, et al. (1995). "Definitive tumor resection and percutaneous bacille Calmette-Guerin for management of renal pelvic transitional cell carcinoma in solitary kidneys." Urology **45**(3): 381-386.

143 [192]. Vegt, P. D., A. P. van der Meijden, et al. (1997). "Does isoniazid reduce side effects of intravesical bacillus Calmette-Guerin therapy in superficial bladder cancer? Interim results of European Organization for Research and Treatment of Cancer Protocol 30911." J Urol **157**(4): 1246-1249.

144 [181]. Witjes, J. A., C. T. Caris, et al. (1998). "Results of a randomized phase III trial of sequential intravesical therapy with mitomycin C and bacillus Calmette-Guerin versus mitomycin C alone in patients with superficial bladder cancer." J Urol **160**(5): 1668-1671; discussion 1671-1662.

145 [34]. Witjes, J. A., J. Palou, et al. (2013). "Current clinical practice gaps in the treatment of intermediate- and high-risk non-muscle-invasive bladder cancer (NMIBC) with emphasis on the use of bacillus Calmette-Guerin (BCG): results of an international individual patient data survey (IPDS)." BJU Int **112**(6): 742-750.

146 [182]. Witjes, J. A., A. P. v d Meijden, et al. (1998). "Long-term follow-up of an EORTC randomized prospective trial comparing intravesical bacille Calmette-Guerin-RIVM and mitomycin C in superficial bladder cancer. EORTC GU Group and the Dutch South East Cooperative Urological Group. European Organisation for Research and Treatment of Cancer Genito-Urinary Tract Cancer Collaborative Group." Urology **52**(3): 403-410.

147 [171]. Witjes, W. P., M. Konig, et al. (1999). "Results of a European comparative randomized study comparing oral bropirimine versus intravesical BCG treatment in BCG-naive patients with carcinoma in situ of the urinary bladder. European Bropirimine Study Group." Eur Urol **36**(6): 576-581.

148 [4]. Yokomizo, A., Y. Kanimoto, et al. (2016). "Randomized Controlled Study of the Efficacy, Safety and Quality of Life with Low Dose bacillus Calmette-Guerin Instillation Therapy for Nonmuscle Invasive Bladder Cancer." J Urol **195**(1): 41-46.

149 [2]. Zeng, S., X. Yu, et al. (2015). "Low-Dose Versus Standard Dose of Bacillus Calmette-Guerin in the Treatment of Nonmuscle Invasive Bladder Cancer: A Systematic Review and Meta-Analysis." Medicine (Baltimore) **94**(49): e2176.

150 [10]. Zhou, X., G. Zhang, et al. (2015). "p53 Status correlates with the risk of recurrence in non-muscle invasive bladder cancers treated with Bacillus Calmette-Guerin: a meta-analysis." PLoS One **10**(3): e0119476.

151 [161]. Zlotta, A. R., J. P. van Vooren, et al. (2000). "What is the optimal regimen for BCG intravesical therapy? Are six weekly instillations necessary?" Eur Urol **37**(4): 470-477.

TOPIC: Transitional Cell Carcinoma or Bladder Cancer or Urothelial Cancer and Combination Therapy

1 [349]. Bachir, B. G., A. Dragomir, et al. (2014). "Contemporary cost-effectiveness analysis comparing sequential bacillus Calmette-Guerin and electromotive mitomycin versus bacillus Calmette-Guerin alone for patients with high-risk non-muscle-invasive bladder cancer." Cancer **120**(16): 2424-2431.

2 [358]. Duchek, M., R. Johansson, et al. (2010). "Bacillus Calmette-Guerin is superior to a combination of epirubicin and interferon-alpha2b in the intravesical treatment of patients with stage T1 urinary bladder cancer. A prospective, randomized, Nordic study." Eur Urol **57**(1): 25-31.

3 [353]. Falke, J., R. J. Lammers, et al. (2013). "Results of a phase 1 dose escalation study of intravesical TMX-101 in patients with nonmuscle invasive bladder cancer." J Urol **189**(6): 2077-2082.

4 [352]. Fernandez-Gomez, J. M., J. Carballido-Rodriguez, et al. (2013). "Treatment of non muscle invasive bladder tumor related to the problem of bacillus Calmette-Guerin availability. Consensus of a Spanish expert's panel. Spanish Association of Urology." Actas Urol Esp **37**(7): 387-394.

5 [350]. Hemdan, T., R. Johansson, et al. (2014). "5-Year outcome of a randomized prospective study comparing bacillus Calmette-Guerin with

epirubicin and interferon-alpha2b in patients with T1 bladder cancer." J Urol **191**(5): 1244-1249.

6 [347]. Noguchi, M., K. Matsumoto, et al. (2016). "An Open-Label, Randomized Phase II Trial of Personalized Peptide Vaccination in Patients with Bladder Cancer that Progressed after Platinum-Based Chemotherapy." Clin Cancer Res **22**(1): 54-60.

7 [351]. Ohyama, C., S. Hatakeyama, et al. (2014). "Neoadjuvant chemotherapy with gemcitabine plus carboplatin followed by immediate radical cystectomy for muscle-invasive bladder cancer." Int J Urol **21**(1): 3-4.

8 [346]. Ojerholm, E., A. Smith, et al. (2017). "Neutrophil-to-lymphocyte ratio as a bladder cancer biomarker: Assessing prognostic and predictive value in SWOG 8710." Cancer **123**(5): 794-801.

9 [357]. Stenzl, A., N. C. Cowan, et al. (2010). "[Update of the Clinical Guidelines of the European Association of Urology on muscle-invasive and metastatic bladder carcinoma]." Actas Urol Esp **34**(1): 51-62.

TOPIC: Transitional Cell Carcinoma or Bladder Cancer or Urothelial Cancer and Interferon

1 [251]. Bazarbashi, S., C. Pai, et al. (2003). "Phase II trial of cisplatin, 5-fluorouracil, and interferon-alpha-2B as first line treatment of advanced urothelial cancer." Urol Oncol **21**(3): 185-189.

2 [231]. Benedict, W. F., M. Fisher, et al. (2014). "Use of monitoring levels of soluble forms of cytokeratin 18 in the urine of patients with superficial bladder cancer following intravesical Ad-IFNalpha/Syn3 treatment in a phase I study." Cancer Gene Ther **21**(3): 91-94.

3 [278]. da Silva, F. C., L. Furtado, et al. (1995). "Comparison of two doses of interferon-alpha-2b in intravesical prophylaxis of superficial bladder tumors. Portuguese Genito-Urinary Group." Eur Urol **28**(4): 291-296.

4 [242]. Liakou, C. I., A. Kamat, et al. (2008). "CTLA-4 blockade increases IFNgamma-producing CD4+ICOShi cells to shift the ratio of effector to regulatory T cells in cancer patients." Proc Natl Acad Sci U S A **105**(39): 14987-14992.

5 [255]. Rajala, P., E. Kaasinen, et al. (2002). "Perioperative single dose instillation of epirubicin or interferon-alpha after transurethral resection for the

prophylaxis of primary superficial bladder cancer recurrence: a prospective randomized multicenter study--FinnBladder III long-term results." J Urol **168**(3): 981-985.

6 [263]. Rajala, P., T. Liukkonen, et al. (1999). "Transurethral resection with perioperative instilation on interferon-alpha or epirubicin for the prophylaxis of recurrent primary superficial bladder cancer: a prospective randomized multicenter study--Finnbladder III." J Urol **161**(4): 1133-1135; discussion 1135-1136.

TOPIC: Transitional Cell Carcinoma or Bladder Cancer or Urothelial Cancer and Ipilimumab

1 [281]. Carthon, B. C., J. D. Wolchok, et al. (2010). "Preoperative CTLA-4 blockade: tolerability and immune monitoring in the setting of a presurgical clinical trial." Clin Cancer Res **16**(10): 2861-2871.

2 [280]. Chen, H., T. Fu, et al. (2014). "CD4 T cells require ICOS-mediated PI3K signaling to increase T-Bet expression in the setting of anti-CTLA-4 therapy." Cancer Immunol Res **2**(2): 167-176.

TOPIC: Transitional Cell Carcinoma or Bladder Cancer or Urothelial Cancer and Nivolumab

1 [339]. Sharma, P., M. K. Callahan, et al. (2016). "Nivolumab monotherapy in recurrent metastatic urothelial carcinoma (CheckMate 032): a multicentre, open-label, two-stage, multi-arm, phase 1/2 trial." Lancet Oncol **17**(11): 1590-1598.

2 [338]. Sharma, P., M. Retz, et al. (2017). "Nivolumab in metastatic urothelial carcinoma after platinum therapy (CheckMate 275): a multicentre, single-arm, phase 2 trial." Lancet Oncol **18**(3): 312-322.

TOPIC: Transitional Cell Carcinoma or Bladder Cancer or Urothelial Cancer and PD1/PD-L1

1 [345]. Carbognin, L., S. Pilotto, et al. (2015). "Differential Activity of Nivolumab, Pembrolizumab and MPDL3280A according to the Tumor Expression of Programmed Death-Ligand-1 (PD-L1): Sensitivity Analysis of Trials in Melanoma, Lung and Genitourinary Cancers." PLoS One **10**(6): e0130142.

TOPIC: Transitional Cell Carcinoma or Bladder Cancer or Urothelial Cancer and Pembrolizumab

1 [340]. Bellmunt, J., R. de Wit, et al. (2017). "Pembrolizumab as Second-Line Therapy for Advanced Urothelial Carcinoma." N Engl J Med **376**(11): 1015-1026.

2 [341]. Plimack, E. R., J. Bellmunt, et al. (2017). "Safety and activity of pembrolizumab in patients with locally advanced or metastatic urothelial cancer (KEYNOTE-012): a non-randomised, open-label, phase 1b study." Lancet Oncol **18**(2): 212-220.

TOPIC: Author Additions

1 [289]. Badalament, R. A., H. W. Herr, et al. (1987). "A prospective randomized trial of maintenance versus nonmaintenance intravesical bacillus Calmette-Guerin therapy of superficial bladder cancer." J Clin Oncol **5**(3): 441-449.

2 [302]. Bohle, A., S. Rusch-Gerdes, et al. (1996). "The effect of lubricants on viability of bacillus Calmette-Guerin for intravesical immunotherapy against bladder carcinoma." J Urol **155**(6): 1892-1896.

3 [303]. Chamie, K., C. S. Saigal, et al. (2011). "Compliance with guidelines for patients with bladder cancer: variation in the delivery of care." Cancer **117**(23): 5392-5401.

4 [290]. Cookson, M. S., H. W. Herr, et al. (1997). "The treated natural history of high risk superficial bladder cancer: 15-year outcome." J Urol **158**(1): 62-67.

5 [304]. Freeman, H. P. (2012). "The origin, evolution, and principles of patient navigation." Cancer Epidemiol Biomarkers Prev **21**(10): 1614-1617.

6 [305]. Freund, K. M., T. A. Battaglia, et al. (2014). "Impact of patient navigation on timely cancer care: the Patient Navigation Research Program." J Natl Cancer Inst **106**(6): dju115.

7 [283]. Gartrell B.A., H. N. M., Hutson T.E., Sonpavde G., Hauke R.J., Starodub A., Small A.C., Tsao C.-K., Galsky M.D. (2012). "Phase II trial of gemcitabine and cisplatin plus ipilimumab as first-line treatment for metastatic urothelial carcinoma." J Clin Oncol **30**(15): Suppl 1.

8 [306]. Gore, J. L., M. S. Litwin, et al. (2010). "Use of radical cystectomy for

patients with invasive bladder cancer." J Natl Cancer Inst **102**(11): 802-811.

9 [307]. Griffiths, T. R. and C. Action on Bladder (2013). "Current perspectives in bladder cancer management." Int J Clin Pract **67**(5): 435-448.

10 [309]. Herr, H. W. (2012). "Intravesical bacillus Calmette-Guerin outcomes in patients with bladder cancer and asymptomatic bacteriuria." J Urol **187**(2): 435-437.

11 [310]. Herr, H. W. and G. Dalbagni (2013). "Intravesical bacille Calmette-Guerin (BCG) in immunologically compromised patients with bladder cancer." BJU Int **111**(6): 984-987.

12 [291]. Hudson, M. A., T. L. Ratliff, et al. (1987). "Single course versus maintenance bacillus Calmette-Guerin therapy for superficial bladder tumors: a prospective, randomized trial." J Urol **138**(2): 295-298.

13 [292]. Jurincic, C. D., U. Engelmann, et al. (1988). "Immunotherapy in bladder cancer with keyhole-limpet hemocyanin: a randomized study." J Urol **139**(4): 723-726.

14 [311]. Kamat, A. M., T. W. Flaig, et al. (2015). "Expert consensus document: Consensus statement on best practice management regarding the use of intravesical immunotherapy with BCG for bladder cancer." Nat Rev Urol **12**(4): 225-235.

15 [293]. Lamm, D., M. Brausi, et al. (2014). "Interferon alfa in the treatment paradigm for non-muscle-invasive bladder cancer." Urol Oncol **32**(1): 35 e21-30.

16 [294]. Lamm, D. L., B. A. Blumenstein, et al. (1991). "A randomized trial of intravesical doxorubicin and immunotherapy with bacille Calmette-Guerin for transitional-cell carcinoma of the bladder." N Engl J Med **325**(17): 1205-1209.

17 [295]. Lamm, D. L., B. A. Blumenstein, et al. (1995). "Randomized intergroup comparison of bacillus calmette-guerin immunotherapy and mitomycin C chemotherapy prophylaxis in superficial transitional cell carcinoma of the bladder a southwest oncology group study." Urol Oncol **1**(3): 119-126.

18 [296]. Lamm, D. L. C., M.; Persad, R.; Soloway, M.; Bohle, A.; Palou, J.; Witjes, J.A.; Akaza, H.; Buckley, R.; Brausi, M. (2008). "Clinical Practice Recommendations for the Management of Non–Muscle Invasive Bladder Cancer." Eur Urol **sup. 7**: 651–666.

19 [297]. Lammers, R. J., W. P. Witjes, et al. (2012). "Intracutaneous and

intravesical immunotherapy with keyhole limpet hemocyanin compared with intravesical mitomycin in patients with non-muscle-invasive bladder cancer: results from a prospective randomized phase III trial." J Clin Oncol **30**(18): 2273-2279.

20 [359]. Massard, C., M. S. Gordon, et al. (2016). "Safety and Efficacy of Durvalumab (MEDI4736), an Anti-Programmed Cell Death Ligand-1 Immune Checkpoint Inhibitor, in Patients With Advanced Urothelial Bladder Cancer." J Clin Oncol **34**(26): 3119-3125.

21 [284]. Matsumoto, K., M. Noguchi, et al. (2011). "A phase I study of personalized peptide vaccination for advanced urothelial carcinoma patients who failed treatment with methotrexate, vinblastine, adriamycin and cisplatin." BJU Int **108**(6): 831-838.

22 [312]. Mengual, L., M. Marin-Aguilera, et al. (2007). "Clinical utility of fluorescent in situ hybridization for the surveillance of bladder cancer patients treated with bacillus Calmette-Guerin therapy." Eur Urol **52**(3): 752-759.

23 [313]. Mohamed, N. E., P. Chaoprang Herrera, et al. (2014). "Muscle invasive bladder cancer: examining survivor burden and unmet needs." J Urol **191**(1): 48-53.

24 [298]. Morales, A., D. Eidinger, et al. (1976). "Intracavitary Bacillus Calmette-Guerin in the treatment of superficial bladder tumors." J Urol **116**(2): 180-183.

25 [288]. Morales, A., H. Herr, et al. (2014). "Efficacy and Safety of MCNA in Patients with Non-Muscle Invasive Bladder Cancer at High-Risk of Recurrence and Progression who Have Failed Treatment with Bacillus Calmette-Guerin." J Urol.

26 [299]. O'Donnell, M. A., J. Krohn, et al. (2001). "Salvage intravesical therapy with interferon-alpha 2b plus low dose bacillus Calmette-Guerin is effective in patients with superficial bladder cancer in whom bacillus Calmette-Guerin alone previously failed." J Urol **166**(4): 1300-1304, discussion 1304-1305.

27 [285]. Powels T, V. N. J., Fine G.D., Eder J.P., Braiteh F.S., Loriot Y., Zambrano C.C., Bellmunt J., Burris H.A., Teng S.-L.M., Shen X., Koeppen H., Hegde P.S., Chen D.S. Petrylak D.P. (2014). "Inhibition of PD-L1 by MPDL3280A and clinical activity in pts with metastatic urothelial bladder cancer (UBC)." J Clin Oncol **32**(15): Suppl 1.

28 [314]. Shah, J. B. and A. M. Kamat (2013). "Strategies for optimizing bacillus Calmette-Guerin." Urol Clin North Am **40**(2): 211-218.

29 [286]. Sharma, P., D. F. Bajorin, et al. (2008). "Immune responses detected in urothelial carcinoma patients after vaccination with NY-ESO-1 protein plus BCG and GM-CSF." J Immunother **31**(9): 849-857.

30 [300]. Shelley, M. D., H. Kynaston, et al. (2001). "A systematic review of intravesical bacillus Calmette-Guerin plus transurethral resection vs transurethral resection alone in Ta and T1 bladder cancer." BJU Int **88**(3): 209-216.

31 [315]. Solsona, E., I. Iborra, et al. (1996). "Extravesical involvement in patients with bladder carcinoma in situ: biological and therapy implications." J Urol **155**(3): 895-899; discussion 899-900.

32 [301]. Tachibana, M., S. Jitsukawa, et al. (1989). "[Comparative study on prophylactic intravesical instillation of bacillus Calmette-Guerin (BCG) and adriamycin for superficial bladder cancers]." Nihon Hinyokika Gakkai Zasshi **80**(10): 1459-1465.

33 [287]. Tanaka, T., H. Kitamura, et al. (2013). "Potential survival benefit of anti-apoptosis protein: survivin-derived peptide vaccine with and without interferon alpha therapy for patients with advanced or recurrent urothelial cancer--results from phase I clinical trials." Clin Dev Immunol **2013**: 262967.

34 [316]. Vedder, M. M., M. Marquez, et al. (2014). "Risk prediction scores for recurrence and progression of non-muscle invasive bladder cancer: an international validation in primary tumours." PLoS One **9**(6): e96849.

35 [317]. Wagner, E. H., E. J. Ludman, et al. (2014). "Nurse navigators in early cancer care: a randomized, controlled trial." J Clin Oncol **32**(1): 12-18.

36 [318]. Witjes, J. A., M. Babjuk, et al. (2014). "Clinical and cost effectiveness of hexaminolevulinate-guided blue-light cystoscopy: evidence review and updated expert recommendations." Eur Urol **66**(5): 863-871.

37 [319]. Xylinas, E., M. Kent, et al. (2013). "Accuracy of the EORTC risk tables and of the CUETO scoring model to predict outcomes in non-muscle-invasive urothelial carcinoma of the bladder." Br J Cancer **109**(6): 1460-1466.
